# Trends in using deep learning algorithms in biomedical prediction systems

**DOI:** 10.3389/fnins.2023.1256351

**Published:** 2023-11-09

**Authors:** Yanbu Wang, Linqing Liu, Chao Wang

**Affiliations:** ^1^School of Strength and Conditioning, Beijing Sport University, Beijing, China; ^2^Department of Physical Education, Peking University, Beijing, China; ^3^Institute of Competitive Sports, Beijing Sport University, Beijing, China

**Keywords:** deep learning, machine learning, bioinformatics, IoT, medical informatics

## Abstract

In the domain of using DL-based methods in medical and healthcare prediction systems, the utilization of state-of-the-art deep learning (DL) methodologies assumes paramount significance. DL has attained remarkable achievements across diverse domains, rendering its efficacy particularly noteworthy in this context. The integration of DL with health and medical prediction systems enables real-time analysis of vast and intricate datasets, yielding insights that significantly enhance healthcare outcomes and operational efficiency in the industry. This comprehensive literature review systematically investigates the latest DL solutions for the challenges encountered in medical healthcare, with a specific emphasis on DL applications in the medical domain. By categorizing cutting-edge DL approaches into distinct categories, including convolutional neural networks (CNNs), recurrent neural networks (RNNs), generative adversarial networks (GANs), long short-term memory (LSTM) models, support vector machine (SVM), and hybrid models, this study delves into their underlying principles, merits, limitations, methodologies, simulation environments, and datasets. Notably, the majority of the scrutinized articles were published in 2022, underscoring the contemporaneous nature of the research. Moreover, this review accentuates the forefront advancements in DL techniques and their practical applications within the realm of medical prediction systems, while simultaneously addressing the challenges that hinder the widespread implementation of DL in image segmentation within the medical healthcare domains. These discerned insights serve as compelling impetuses for future studies aimed at the progressive advancement of using DL-based methods in medical and health prediction systems. The evaluation metrics employed across the reviewed articles encompass a broad spectrum of features, encompassing accuracy, precision, specificity, F-score, adoptability, adaptability, and scalability.

## 1. Introduction

Deep learning (DL) algorithms have revolutionized various fields by enabling machines to learn and make intelligent decisions from complex and large-scale data (Li et al., 2019). Their importance lies in their ability to automatically extract meaningful features and patterns from data, leading to advanced capabilities in areas such as computer vision, natural language processing (NLP), and data analysis (Alber et al., 2019). DL algorithms excel in tasks that involve recognizing and understanding intricate patterns, making them particularly valuable in domains such as medical imaging analysis, autonomous driving, voice recognition, and recommendation systems (Kocheturov et al., 2019; Amiri et al., 2023). They have the potential to uncover hidden insights, improve accuracy, and drive innovation across industries, enabling breakthroughs in healthcare, finance, manufacturing, and more (Aldahiri et al., 2021). The power of DL algorithms lies in their capacity to process vast amounts of data and learn complex representations, making them a driving force in the advancement of AI and shaping the future of technology (Kumari and Krishna, 2018).

Biomedical prediction systems play a crucial role in healthcare by providing valuable insights and predictions that aid in disease diagnosis, prognosis, treatment planning, and patient management (Yi et al., 2022). These systems leverage advanced machine learning (ML) and statistical techniques to analyze vast amounts of biomedical data, including patient records, medical images, genetic information, and clinical measurements (Ahmad Mir et al., 2020). By integrating and analyzing this diverse data, biomedical prediction systems can identify patterns, detect early signs of diseases, predict patient outcomes, and support evidence-based decision-making by healthcare professionals (Shafik et al., 2020). These systems have the potential to improve patient care, optimize treatment strategies, reduce healthcare costs, and ultimately save lives (Wang et al., 2018). They enable personalized medicine, facilitate precision healthcare, and contribute to advancements in medical research, making them indispensable tools in the pursuit of better health outcomes and improved patient wellbeing. The use of DL algorithms in medical image prediction systems holds significant importance in the field of healthcare (Khan et al., 2020). Medical images, such as X-rays, MRI scans, and CT scans, contain a wealth of information that can assist in accurate diagnosis, treatment planning, and monitoring of various medical conditions (Mittal and Hasija, 2020). DL algorithms, particularly convolutional neural networks (CNNs), have demonstrated remarkable capabilities in extracting intricate patterns and features from these images, enabling more precise and automated analysis (Javan et al., 2018). One key advantage of DL algorithms in medical image prediction systems is their ability to learn directly from raw pixel data, eliminating the need for manual feature extraction. This not only saves time but also enhances the accuracy and efficiency of image interpretation (Meiring et al., 2018). DL models can automatically detect subtle abnormalities, classify different tissue types, segment organs, and even predict the progression of diseases (Chakraborty and Mali, 2023). By leveraging large datasets and training on diverse medical images, DL algorithms can improve the accuracy of predictions and assist healthcare professionals in making informed decisions (Kumar and Mahajan, 2020). They can aid in the early detection of diseases, allowing for timely intervention and potentially saving lives. Moreover, these algorithms have the potential to reduce human error and variability in image interpretation, leading to more consistent and reliable diagnoses. Additionally, DL algorithms enable the integration of medical image analysis with other clinical data, such as patient history and genetic information, to provide a comprehensive understanding of individual patients (Shinde and Rajeswari, 2018). This personalized approach enhances treatment planning, enables tailored therapies, and supports precision medicine (Verma et al., 2019). Overall, the use of DL algorithms in medical image prediction systems has the potential to revolutionize healthcare by enhancing diagnostic accuracy, improving patient outcomes, and driving advancements in medical research. It enables efficient and automated analysis of medical images, paving the way for more precise and personalized care for patients.

To the best of our knowledge, no study has yet assessed and thoroughly analyzed all of the comprehensive DL methods used for medical health prediction systems. However, we have conducted a consolidated SLR to assess DL methods used in medical health prediction systems. As a result, our study has focused on employing DL approaches as the fundamental background in climate change mitigation. The significance of this work stems from its in-depth examination of different successful DL methods used to map health prediction system issues. An SLR is used in this article to test, incorporate, and analyze solutions for the same publications. Furthermore, we have divided DL method categories into six groups: CNNs, recurrent neural networks (RNNs), generative adversarial networks (GANs), long short-term memory (LSTM) models, support vector machines (SVMs), and hybrid methods. We have investigated several aspects of each category, including benefits, downsides, simulation environment, dataset, and the DL approach used in applications. This research has looked into the methodology and applications of DL methods in medical and health prediction systems, as well as a variety of suggested ways to use them in medical and health prediction systems. In addition, we have looked at future work that will be required in future research. So, this study’s contributions are as follows:

- Touching on a comprehensive survey of the present challenges related to DL methods used in medical and health prediction systems;
- Proposing a systematic overview of the existing DL methods used in medical and health prediction systems in studied research;
- Assessing each area that has customized DL methods with various insights such as benefits, limitations, datasets, security involvement, and simulation stings;
- Outlining the pivotal sides that drive the referred methods to improve in future studies;
- Illustrating the descriptions of particular DL methods utilized in different research.

The following classification creates the framework of this study. The next section delves deeper into the primary perspectives and proper terminology of DL techniques for health prediction systems. Part 3 looks at the survey articles that are related to it. The research methods and mechanisms for article selection are covered in Part 4. Part 5 contains the articles that have been chosen for research and analysis. The next part contains a comparison and debate, which is expanded in Part 6. Finally, in Part 7, open issues are asserted and the consequences are expressed.

## 2. Fundamental concepts and terminology

DL algorithms have shown great promise in the field of medical image analysis. These algorithms are a subset of ML techniques that are inspired by the structure and function of the human brain, specifically neural networks. They are designed to automatically learn and extract meaningful features from complex medical images, such as X-rays, MRI scans, CT scans, and histopathology slides. CNN is the most commonly used DL algorithm for medical image analysis. CNNs are composed of multiple layers of interconnected nodes, called neurons, which mimic the behavior of visual neurons in the brain. These networks can automatically learn hierarchical representations of features from raw pixel data by applying a series of convolutional, pooling, and activation operations. CNNs are highly effective in capturing spatial information, identifying patterns, and distinguishing between different image regions.

In medical image analysis, DL algorithms play a crucial role in various tasks. They can perform image classification, where they classify images into different categories or identify specific diseases or conditions. They can also perform object detection, locating and outlining specific structures or abnormalities within an image. Image segmentation is another important task, where DL algorithms can separate different regions of an image, such as organs or tumors, to enable precise analysis and measurement. DL algorithms can also be used for image generation, such as generating synthetic medical images or enhancing the resolution of low-quality images. The power of DL algorithms lies in their ability to automatically learn complex patterns and features directly from large-scale medical image datasets. They can adapt and improve their performance through training on diverse examples, enabling them to generalize well to new and unseen data. This capability makes them particularly valuable in medical image analysis, where large and diverse datasets are available for training. Overall, DL algorithms have revolutionized the field of medical image analysis by enabling more accurate and efficient interpretation of complex medical images. They have the potential to enhance diagnostics, improve treatment planning, and support clinical decision-making, ultimately leading to better patient care and outcomes.

Prediction systems for medical images using DL involve the utilization of DL algorithms to analyze and interpret medical images for predictive purposes. These systems employ advanced neural network architectures, such as CNN and RNN, to extract meaningful features from the images and make accurate predictions. One common approach is image classification, where DL models are trained on large datasets of labeled medical images to classify images into different categories, such as normal or abnormal, or to identify specific diseases or conditions. Another method is object detection and localization, where DL models are trained to detect and outline regions of interest within the images, such as tumors or anatomical structures. Additionally, segmentation techniques can be employed to precisely delineate and separate different structures or pathologies within the images. These prediction systems enable improved diagnosis, prognosis, and treatment planning, ultimately leading to better patient care and outcomes in the field of medical imaging.

The effects of DL algorithms in medical image prediction systems are significant and transformative. DL algorithms have the potential to revolutionize medical imaging by enabling more accurate and efficient analysis of medical images, leading to improved diagnostic accuracy and patient outcomes. There are some key effects of DL algorithms in medical image prediction systems. DL algorithms have shown remarkable performance in image analysis tasks, surpassing traditional methods in accuracy. They can effectively learn complex patterns and features in medical images, allowing for more precise and reliable predictions. In addition, DL algorithms can process large volumes of medical images in a relatively short amount of time, enabling faster and more efficient analysis. This can be particularly beneficial in time-sensitive scenarios, such as emergencies, where quick and accurate diagnosis is crucial. Moreover, DL algorithms enable the automation of image analysis tasks, reducing the dependence on human expertise and subjective interpretation. This leads to increased standardization and consistency in medical image interpretation across different healthcare providers. It is worth mentioning that DL algorithms can aid in the early detection and diagnosis of various diseases and conditions, including cancer, neurological disorders, and cardiovascular diseases (Zemouri et al., 2018; Baltres et al., 2020). They can detect subtle abnormalities or features that might be missed by human observers, enabling timely intervention and treatment. Moreover, DL algorithms have the potential to facilitate personalized medicine by leveraging large-scale medical image datasets and patient-specific information. They can assist in tailoring treatment plans and predicting patient outcomes based on individual characteristics and image-based biomarkers. In addition, DL algorithms contribute to the advancement of medical research by enabling the analysis of large-scale datasets and the extraction of meaningful insights. They can assist in discovering new imaging biomarkers, uncovering relationships between image features and clinical outcomes, and supporting the development of novel diagnostic and therapeutic approaches. In summary, DL algorithms have a profound impact on medical image prediction systems, offering improved accuracy, efficiency, automation, and personalized insights. They have the potential to revolutionize the field of medical imaging, enhancing diagnosis, treatment planning, and patient care.

In summary, DL algorithms, notably CNNs, excel in medical image analysis, particularly in tasks such as classification and segmentation. They extract crucial features from complex images such as X-rays and MRI scans, leading to more precise interpretation. In predictive systems, DL algorithms enhance diagnosis and treatment planning, reduce reliance on human interpretation, and enable personalized medicine, ultimately revolutionizing medical imaging for better patient care.

### 2.1. Importance of biomedical prediction systems using DL algorithms

Health prediction systems that utilize DL algorithms offer several important benefits and hold significant importance in healthcare. Here are some key reasons why health prediction systems using DL algorithms are crucial (Ghazal et al., 2022). Health prediction systems can aid in the early detection of diseases. DL algorithms can analyze a wide range of patient data, such as medical records, genetic information, and imaging scans, to identify patterns and indicators that may signify the presence of a disease (Hosseini et al., 2016). Early detection allows for timely interventions, leading to better treatment outcomes and potentially saving lives. Also, DL-based prediction systems enable personalized medicine approaches by considering individual patient characteristics, genetics, and health records (Zampieri et al., 2019). By analyzing diverse data sources, these systems can predict the risk of disease development, progression, or recurrence for a specific patient. This information can guide healthcare professionals in tailoring treatment plans and interventions to the unique needs of each patient, improving patient outcomes and reducing healthcare costs (Papadakis et al., 2019). DL algorithms can assist healthcare professionals in treatment planning and decision-making. By analyzing patient data, including clinical records, medical imaging, and genetic profiles, DL models can provide insights into optimal treatment options, predict treatment response, and estimate potential side effects or complications (Ali et al., 2019). This information can support clinicians in making well-informed decisions, improving treatment efficacy, and reducing the risk of adverse events. Prognostic Assessment: Health prediction systems using DL algorithms can offer valuable prognostic assessments. By analyzing patient data longitudinally, these systems can predict disease progression, identify potential complications, and estimate patient outcomes (Vinitha et al., 2018). This information enables healthcare providers to offer personalized prognostic information to patients and develop appropriate management plans accordingly. As well, DL-based prediction systems can assist in efficient resource allocation and population health management. By analyzing large-scale health data, such as population demographics, disease prevalence, and risk factors, these systems can predict the future healthcare needs of specific populations (Kirola et al., 2022). This information can guide policymakers and healthcare organizations in allocating resources, planning preventive interventions, and designing targeted public health campaigns to improve population health outcomes. Additionally, DL algorithms in health prediction systems can contribute to scientific advancements and research endeavors. By analyzing vast amounts of data, DL models can identify novel biomarkers, uncover complex disease mechanisms, and generate hypotheses for further investigation (Li et al., 2019). These insights can drive discoveries, improve scientific understanding, and pave the way for innovative treatments and interventions. Overall, health prediction systems using DL algorithms have the potential to transform healthcare by facilitating early detection, enabling personalized medicine, guiding treatment decisions, improving patient outcomes, optimizing resource allocation, and driving scientific advancements. These systems can enhance healthcare delivery, contribute to precision medicine approaches, and ultimately lead to improved patient care and wellbeing (Zhang et al., 2021).

### 2.2. DL application in medical prediction systems

DL is a subset of ML that focuses on training artificial neural networks (ANNs) with multiple layers to learn and extract features from complex data representations. DL has found a wide range of applications across various domains due to its ability to handle large-scale datasets and automatically learn hierarchical representations (Yu and Jiang, 2020). DL has found wide-ranging applications across various domains. In computer vision, DL models excel in tasks such as object detection, image classification, and facial recognition, impacting areas such as autonomous vehicles and medical imaging. In natural language processing, DL enables tasks such as translation, sentiment analysis, and speech recognition, driving advancements in chatbots and virtual assistants. DL’s influence also extends to recommendation systems in e-commerce and streaming platforms. Additionally, it has made significant strides in finance, healthcare, robotics, and creative fields, showcasing its adaptability and potential in tackling real-world challenges.

DL has found wide-ranging applications across various domains. In computer vision, DL models excel in tasks such as object detection, image classification, and facial recognition, impacting areas such as autonomous vehicles and medical imaging. In natural language processing, DL enables tasks such as translation, sentiment analysis, and speech recognition, driving advancements in chatbots and virtual assistants. DL’s influence also extends to recommendation systems in e-commerce and streaming platforms. Additionally, it has made significant strides in finance, healthcare, robotics, and creative fields, showcasing its adaptability and potential in tackling real-world challenges. DL is transforming biomedical prediction systems by efficiently analyzing complex datasets. DL models excel in disease diagnosis, drug discovery, genomics, and medical imaging. They analyze patient data, enabling early detection and personalized treatment recommendations. DL also revolutionizes drug discovery and enhances genomics analysis. In medical imaging, DL rivals human expertise in anomaly detection and outcome prediction. These applications promise to revolutionize healthcare with more accurate diagnoses, personalized treatments, and improved drug discovery.

### 2.3. DL algorithm usages in biomedical prediction systems and DL mechanisms in general

DL algorithms, driven by multi-layered neural networks, play a vital role in biomedical prediction systems, leveraging their capacity to handle complex datasets. They excel in tasks such as disease classification, diagnosis, and drug discovery. In medical imaging, DL models accurately identify abnormalities and assist in interpretation. Additionally, they enhance prognostic assessments based on patient data analysis. DL’s effectiveness arises from its use of ANNs, which process data through interconnected layers, learning increasingly intricate representations. Backpropagation, a key training step, adjusts network weights based on predicted and actual outputs, facilitating learning from labeled data. Optimization algorithms such as stochastic gradient descent further refine the model’s performance. Overall, DL is a pivotal tool in biomedical applications, offering precise insights and solutions across various domains.

## 3. Relevant reviews

DL methods are recognized as groundbreaking techniques for proposing innovative ideas in medical and health prediction systems. Gupta and Katarya (2020) provided a comprehensive analysis of the existing research on utilizing social media platforms for healthcare surveillance with the aid of ML techniques. The authors conducted a systematic review to identify and evaluate relevant studies in this domain. They identified key themes and trends, such as the utilization of NLP and sentiment analysis algorithms for disease surveillance, public health monitoring, adverse event detection, and predicting health-related behaviors. The review highlighted the potential benefits of social media-based surveillance systems, such as their ability to provide real-time data and capture diverse population perspectives. However, the authors also emphasized the challenges and limitations, such as ensuring data quality, addressing privacy concerns, and handling the vast amount of information generated. Overall, their article contributed valuable insights into the use of ML in social media-based healthcare surveillance and provides recommendations for future research directions in this rapidly evolving field.

In addition, Bzdok and Ioannidis (2019) addressed the challenges and opportunities in understanding complex biological systems through exploration, inference, and prediction. The authors discussed the importance of exploring diverse data sources, such as high-throughput genomics and neuroimaging, to uncover meaningful patterns and relationships. They highlighted the significance of inference methods, such as statistical modeling and network analysis, in extracting meaningful information from large-scale datasets. Additionally, their article emphasized the value of predictive modeling to forecast future outcomes and make informed decisions in neuroscience and biomedicine. The authors also discussed the need for interdisciplinary collaborations and the integration of computational approaches to tackle the complexity of biological systems. Their article provided a comprehensive overview of the current state of exploration, inference, and prediction in these fields, underscoring the need for advancing methodologies and fostering interdisciplinary research to advance our understanding of the brain and biomedical processes.

Furthermore, Pugliese et al. (2021) offered a comprehensive analysis of the current landscape of ML in various domains. The authors present global trends in ML research, highlighting the increasing adoption of this approach across industries such as healthcare, finance, and transportation. They discussed emerging research directions, including DL, reinforcement learning, and interpretability of ML models. Their article also addressed regulatory aspects, emphasizing the importance of ethical considerations, transparency, and accountability in deploying ML systems. The authors provided insights into regulatory frameworks and guidelines implemented by different countries and organizations to ensure the responsible and ethical use of ML technologies. This article provided valuable insights into the global trends, research directions, and regulatory considerations in the field of ML, shedding light on its growing impact and the need for responsible implementation.

Moreover, Kourou et al. (2015) explored the utilization of ML techniques in the field of cancer research for prognosis and prediction purposes. The authors discussed the significance of accurate cancer prognosis and prediction in determining appropriate treatment plans and improving patient outcomes. They highlighted various ML approaches employed in this context, including SVMs, random forests, neural networks, and DL algorithms. Their article provided an overview of different data sources used, such as gene expression data, genomic sequencing data, and medical imaging data, and discussed the challenges associated with data preprocessing and feature selection. The authors presented several case studies where ML models have been successfully applied for cancer prognosis and prediction, covering a range of cancer types including breast, lung, and prostate cancer. They also discussed the limitations and prospects of ML in this field, emphasizing the importance of interpretability, robustness, and the integration of multi-omics data for improved accuracy. Their article showcased the potential of ML as a valuable tool in cancer research for prognosis and prediction, highlighting its ability to analyze complex data and provide personalized insights for better clinical decision-making.

In addition, (Singh et al., 2020) explored the use of AI and ML techniques in the field of biomedical material design for predicting toxicity. The authors highlighted the significance of developing safe and effective biomedical materials and discussed the challenges associated with traditional experimental methods for toxicity assessment. They presented various AI and ML approaches that have been employed to predict the toxicity of biomedical materials, including the use of molecular descriptors, structure–activity relationship models, and DL algorithms. Their article discussed the advantages of using AI and ML in this context, such as the ability to analyze large datasets, extract meaningful patterns, and make accurate predictions. The authors also addressed the limitations and future directions of AI and ML in biomedical material design, emphasizing the importance of data quality, model interpretability, and regulatory considerations. Their article highlighted the potential of AI and ML in empowering advanced biomedical material design by providing efficient and reliable methods for toxicity prediction, contributing to the development of safer and more effective materials for various biomedical applications. Table 1 depicts a summary of related works. In summary, in this section, we discussed various medical research studies utilizing ML and DL techniques. Gupta and Katarya review social media’s role in healthcare surveillance, highlighting advantages and challenges. Bzdok and Ioannidis emphasize diverse data sources and inference methods in understanding complex biological systems, while Pugliese et al. cover global ML trends and regulatory aspects. Kourou et al. focus on ML’s potential in personalized cancer research and (Bzdok and Ioannidis, 2019) explore predicting toxicity in biomedical materials, stressing data quality and model interpretability.

**Table 1:** Summary of related works.

Authors	Main idea	Advantage	Disadvantage
Gupta and Katarya (2020)	Providing a comprehensive analysis of the existing research on utilizing social media platforms for healthcare surveillance with the aid of ML techniques	Contributing valuable insights into the use of ML in social media-based healthcare surveillance	Some parameters for differentiating between investigated methods have been overlooked
Bzdok and Ioannidis (2019)	Addressing the challenges and opportunities in understanding complex biological systems through exploration, inference, and prediction	Providing a comprehensive overview of the current state of exploration, inference, and prediction in these fields	Lack of appropriate schematic comparison between methods
Pugliese et al. (2021)	Offering a comprehensive analysis of the current landscape of ML in various domains	Providing valuable insights into global trends, research directions	Some parameters for differentiating between methods have been overlooked
Kourou et al. (2015)	Exploring the utilization of ML techniques in the field of cancer research for prognosis and prediction purposes	Presented several case studies where ML models have been successfully applied for cancer prognosis and prediction, covering a range of cancer types including breast, lung, and prostate cancer	Lack of integration between sections
Singh et al. (2020)	Exploring the use of AI and ML techniques in the field of biomedical material design for predicting toxicity	Highlighting valuable comparisons between methods comprehensively	Some parameters for differentiating between methods have been overlooked
Our work	Proposing a novel taxonomy of DL methods used in medical healthcare prediction systems	Presenting a comprehensive insight toward DL-based methods in medical healthcare prediction systems from various aspects	Lack of accessibility to non-English-language articles

## 4. Methodology of research

We scrutinized relevant documents that, to some extent, investigated the use of deep symmetry in medical image segmentation. Employing the SLR methodology, this section subsequently comprehends the image segmentation field. The SLR technique involves a comprehensive assessment of all research conducted on a significant topic. This section concludes an extensive exploration of ML techniques in the image segmentation realm. Additionally, the dependability of the research selection methods is scrutinized. In the subsequent subsections, we have provided supplementary information concerning research techniques, encompassing the selection metrics and research inquiries.

### 4.1. Question formalization

The main objectives of the research are to identify, evaluate, and distinguish the important articles in the field of image segmentation in ML applications. To achieve these goals, a systematic literature review (SLR) can be employed to thoroughly examine the components and features of methods used in this context. Additionally, an SLR helps in gaining a deep understanding of the key challenges and difficulties associated with this area. The following paragraph outlines several research inquiries:

Research Question 1: *In what manner can ML techniques in the field of medical and health prediction systems be categorized? The answer to this question can be found in Part 5.*

Research Question 2: *What types of techniques do scholars employ to execute their investigation? Parts 5.1 to 5.7 elucidate this query.*

Research Question 3: *Which parameters attracted the most attention in the papers? What are the most popular DL methods utilized in medical and health prediction systems? The answer to this question is included in Part 6.*

Research Question 4: *What unexplored prospects exist in this area? Part 7 proffers the answer to this question.*

### 4.2. Paper selection process

**Figure 1:**
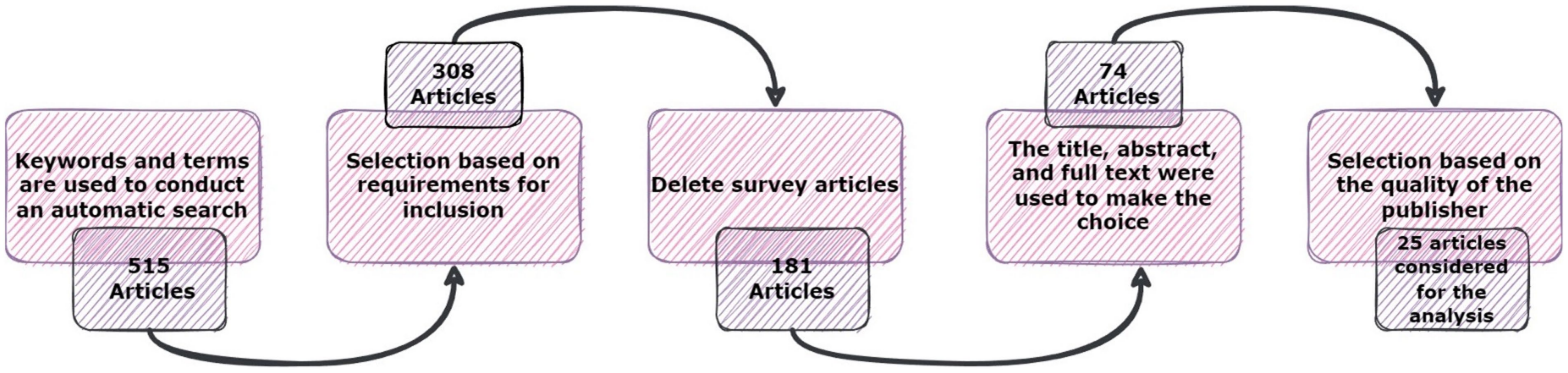
The phases of the article searching and selection process.

**Figure 2:**
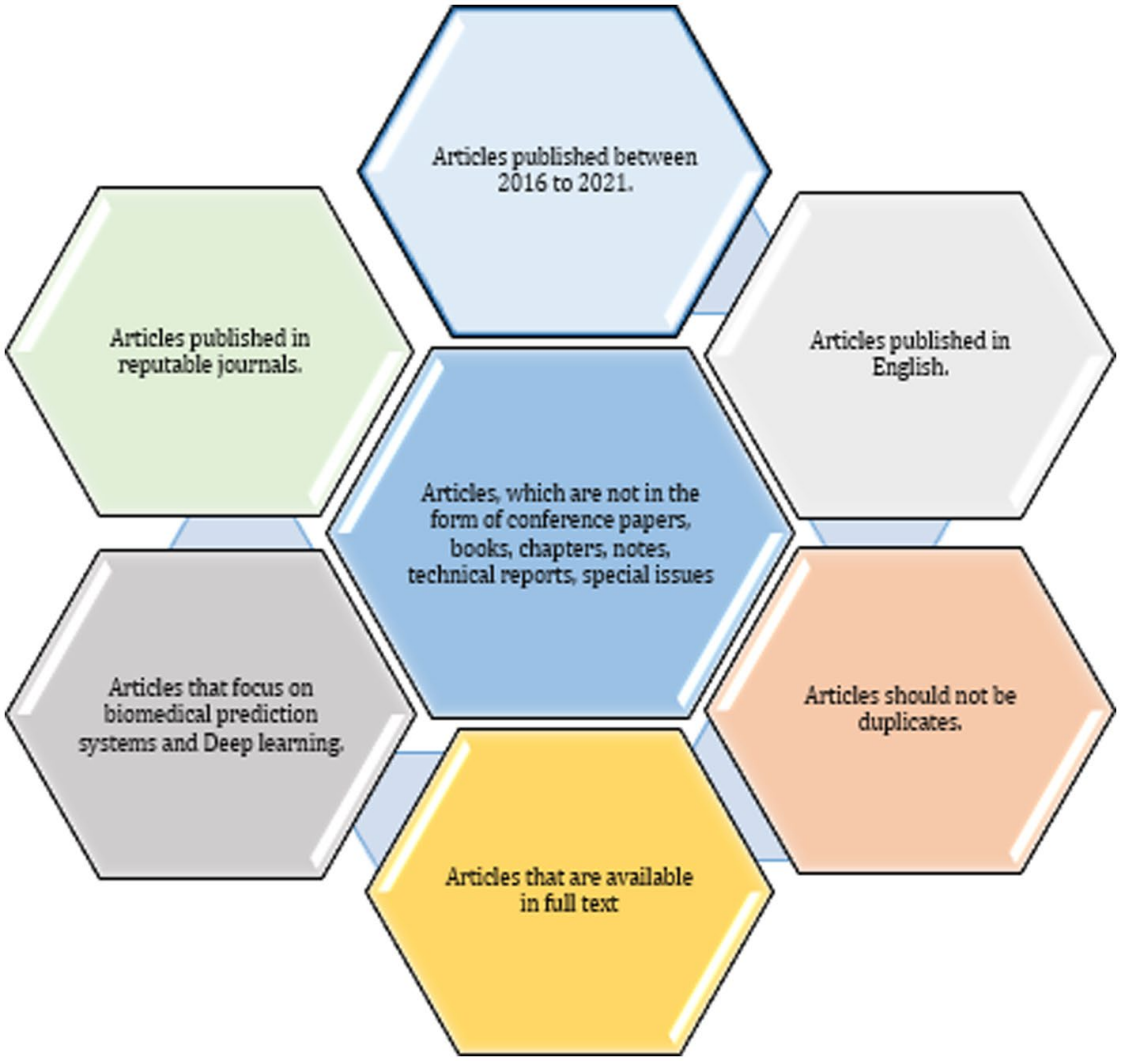
Criteria for inclusion in the paper selection process.

**Figure 3:**
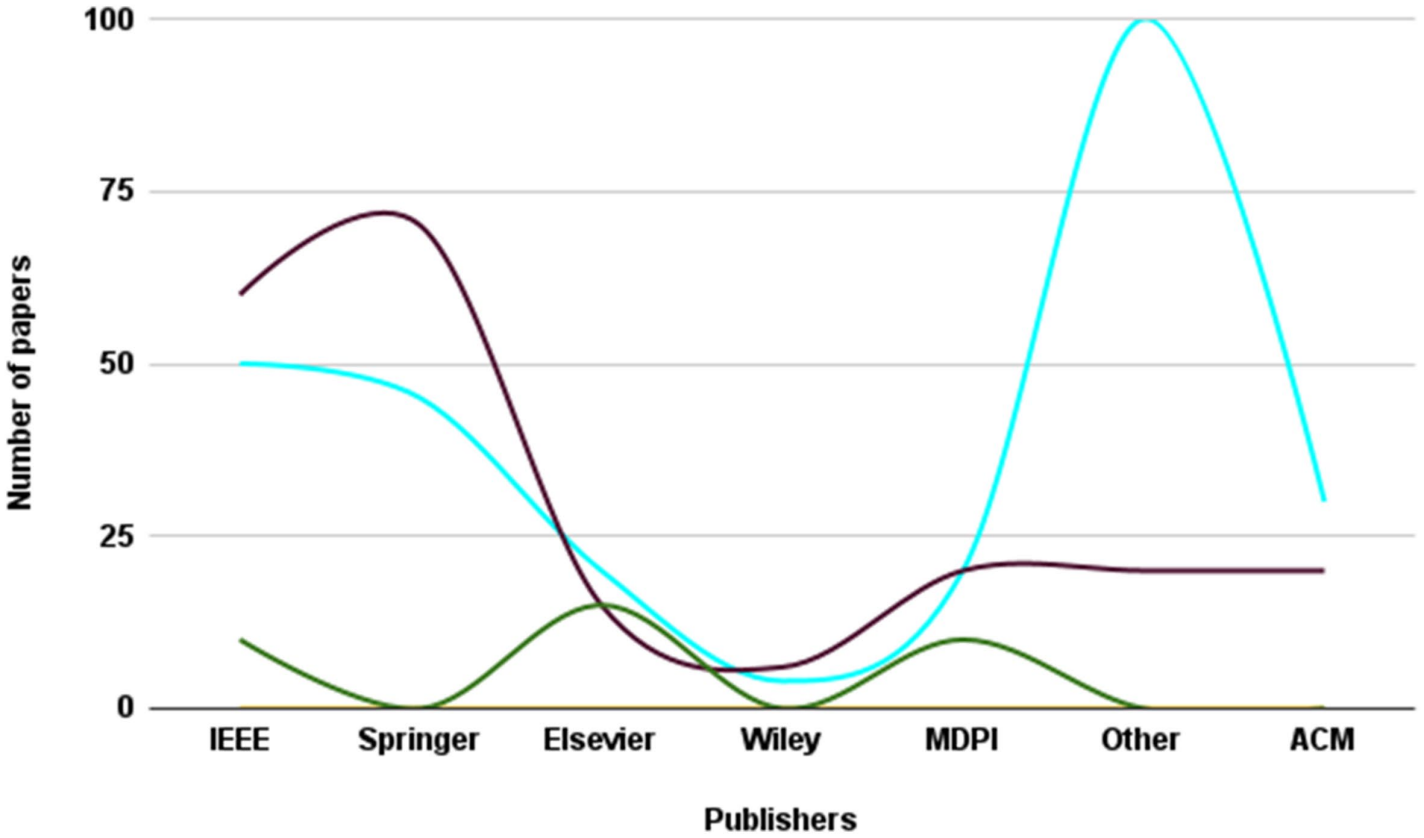
Frequency of publications of studied paper in first stage of paper selection.

The present investigation’s pursuit and selection methodologies are classified into four distinct phases, as depicted in Figure 1. In the initial phase, a comprehensive list of keywords and phrases was utilized to scour various sources, as demonstrated in Table 2. An electronic database was employed to retrieve relevant documents, including chapters, journals, technical studies, conference papers, notes, and special issues, resulting in a total of 515 articles. These articles were then subjected to an exhaustive analysis based on a set of predetermined standards, and only those meeting the stipulated criteria, illustrated in Figure 2, were selected for further evaluation. The distribution of publishers in this initial phase is shown in Figure 3, and the number of articles left after the first phase was 308.

**Table 2:** Keywords and search criteria.

S#	Keywords and search criteria	S#	Keywords and search criteria
S1	“DL” and Healthcare”	S6	“AI” and “Healthcare”
S2	“ML” and “Medical prediction systems”	S7	“Healthcare” and “DL-based methods”
S3	“DL” and “Medical prediction systems”	S8	“DL” and “Medical Healthcare”
S4	“AI” and “Medical and Health prediction Systems”	S9	“DL methods” and “Prediction Systems”

**Figure 4:**
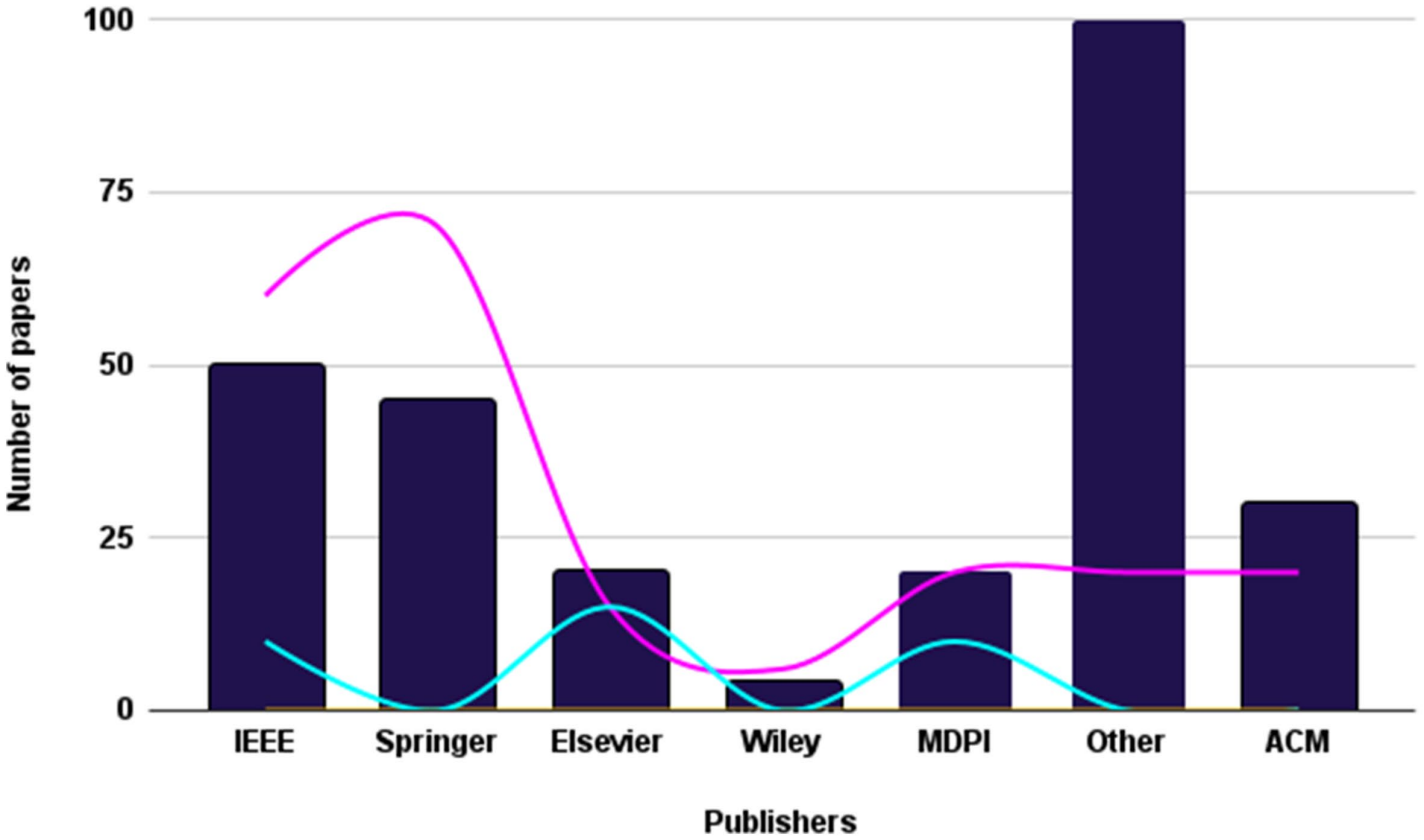
Frequency of publications of studied paper in second stage of paper selection.

**Figure 5:**
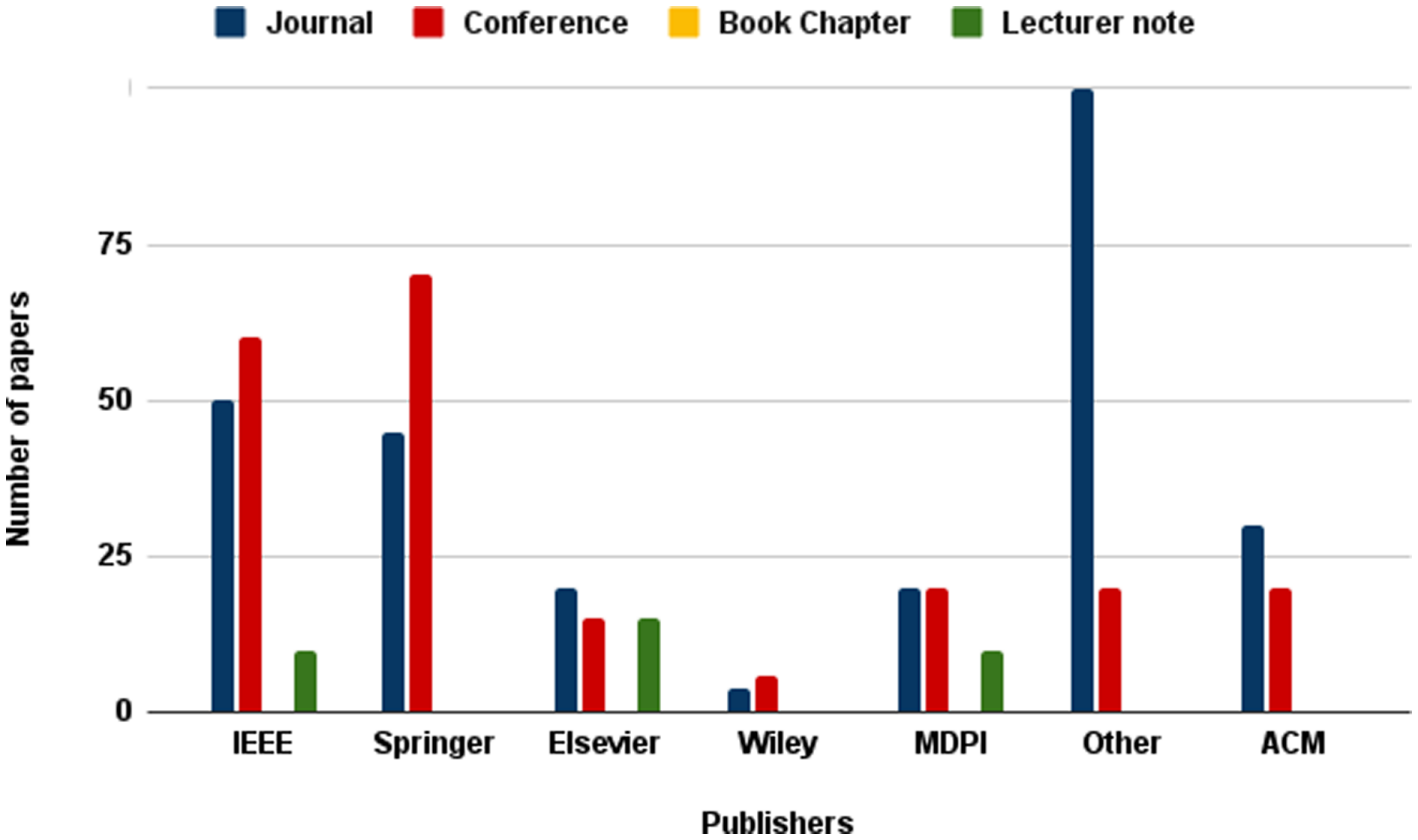
Frequency of publications of studied paper in third stage of paper selection.

**Figure 6:**
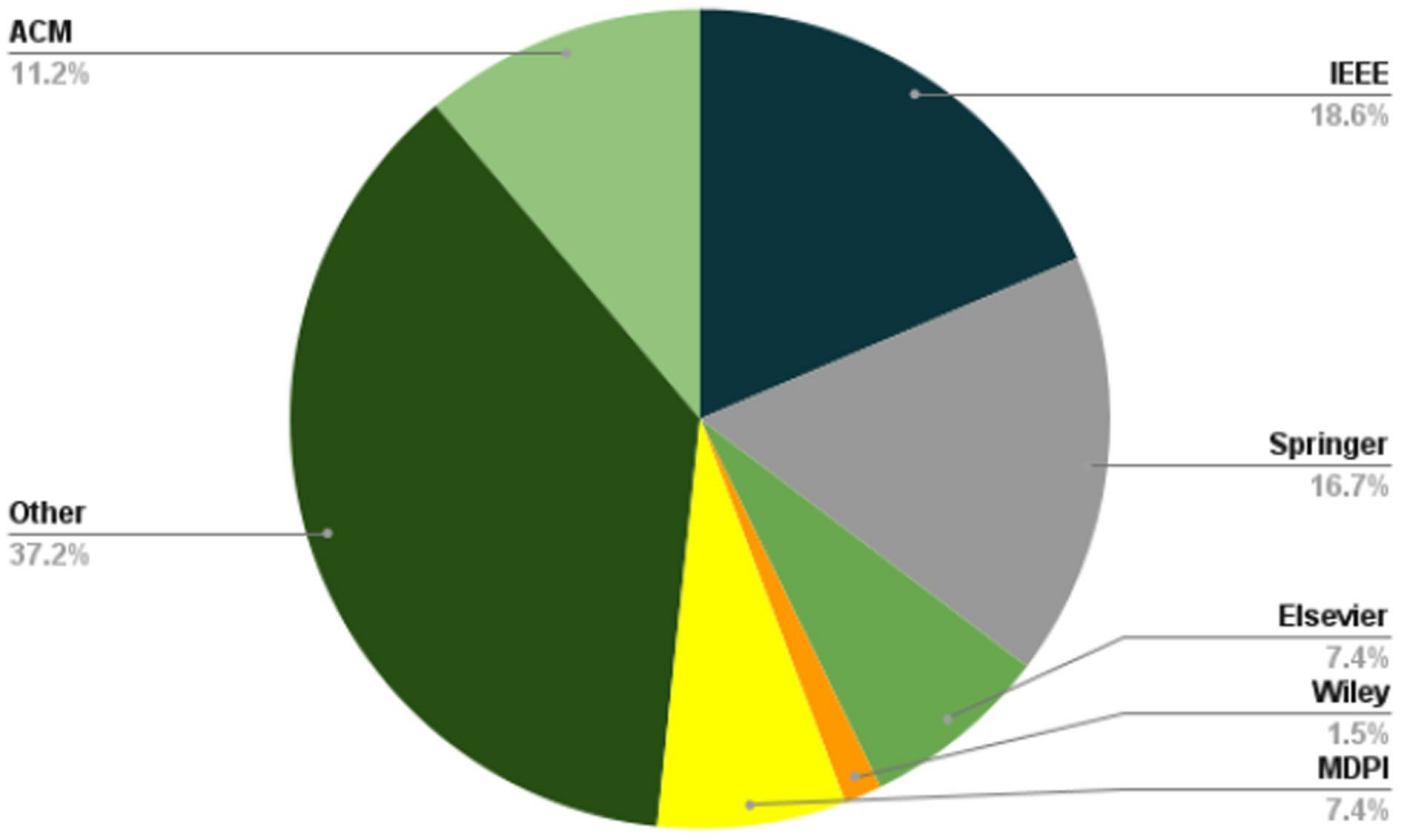
Frequency of publications of studied paper in second stage of paper selection.

During the next stage, a detailed examination of the titles and abstracts of the chosen articles was carried out, with a specific focus on their discussion, methodology, analysis, and conclusion, to ensure their alignment with the study’s objectives. As depicted in Figure 4, only 181 articles remained after this initial screening. The aim was to choose articles that met the predetermined criteria set for the study. After careful deliberation, 74 articles were manually selected to explore additional publications related to Figure 5. For the last step, as depicted in Figure 6, 30 articles were left and selected for the final phase of the study.

## 5. ML/DL algorithms for biomedical prediction systems

**Figure 7:**
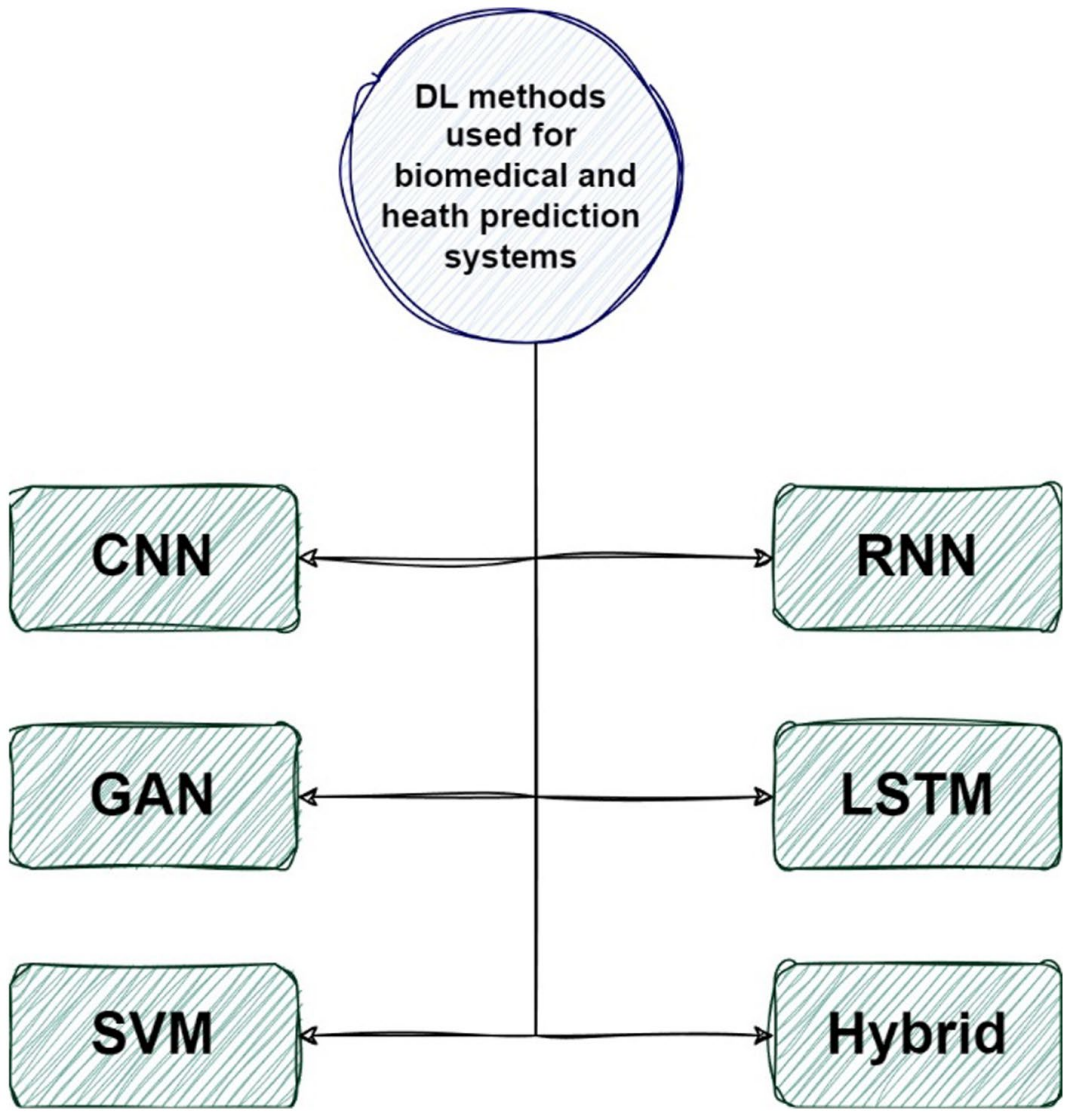
The proposed taxonomy of Bioinformatics.

The role of the CNN method in DL-based algorithms in biomedical prediction systems is to effectively analyze complex biomedical data, extract relevant features, and enable accurate predictions for tasks such as disease diagnosis, medical imaging analysis, and genomic sequence analysis. In this section, we explore the DL methods in medical and healthcare prediction systems. We will present a collection of 30 articles that meet our selection criteria. Initially, we categorize the techniques into six main groups, CNNs, RNNs, GANs, LSTMs, SVMs, and hybrid methodologies, which encompass various approaches. The taxonomy proposed for DL-based methods in medical and health prediction systems is illustrated in Figure 7.

### 5.1. Convolutional neural network algorithms for biomedical prediction systems

In this regard, Jena et al. (2022) investigated the influence of different parameters on the performance of a DL model for diabetic retinopathy classification. The authors explored the effects of varying network architecture, activation functions, dropout rates, and learning rates on the model’s accuracy. Based on their findings, they proposed a novel 2-branch CNN architecture specifically designed for diabetic retinopathy classification. They also presented a case study that demonstrates the integration of this CNN model into an Internet of Things (IoT)–Blockchain-based smart healthcare system, showcasing its potential in real-world applications. Their article provided valuable insights into the impact of parametric variations on DL models for medical image classification and offered a promising approach for diabetic retinopathy detection within a smart healthcare framework.

In addition, Azimi et al. (2018) explored the integration of hierarchical edge-based DL techniques into healthcare IoT systems. The authors highlighted the growing demand for efficient and reliable healthcare systems and the potential of IoT technologies in transforming healthcare delivery. They proposed a hierarchical edge computing architecture that leverages DL models at different levels to enable real-time and context-aware analysis of healthcare data. Their article discussed the challenges faced in healthcare IoT systems, such as limited bandwidth, privacy concerns, and latency issues, and presented a solution using edge computing to overcome these challenges. The authors also provided an investigation of various DL models, including CNN, RNN, and GAN, and discussed their application in healthcare IoT scenarios. They highlighted the advantages of using edge-based DL, such as reduced communication overhead, improved privacy, and faster response times. Their article emphasized the potential of hierarchical edge-based DL in empowering healthcare IoT systems by enabling intelligent data processing, real-time decision-making, and improved patient care.

Moreover, Lv et al. (2022) presented a novel approach for smart healthcare using DL-based predictive evaluation. The authors focused on the integration of multimedia technologies and DL algorithms to enable interactive and intelligent healthcare services. They highlighted the need for personalized and context-aware healthcare systems that can adapt to individual patient needs. The proposed framework utilizes DL techniques, such as CNN and RNN, to analyze multimedia data including images, videos, and sensor readings. These models are trained on large datasets to learn patterns and relationships, enabling accurate prediction and evaluation of healthcare conditions. The authors discussed the benefits of using DL in smart healthcare, such as improved accuracy, real-time analysis, and scalability. They also addressed the challenges and limitations of the proposed approach, including data privacy concerns, model interpretability, and computational complexity. Their article highlighted the potential of DL-based predictive evaluation in revolutionizing smart healthcare systems by providing personalized, proactive, and efficient healthcare services.

In addition, Ismail et al. (2020) presented a novel approach for analyzing regular health factors in an Internet-of-Medical Things (IoMT) environment using CNN. The authors aimed to develop a robust and efficient model that can analyze health-related data collected from various IoMT devices. They proposed a CNN-based architecture that takes into account multiple health factors such as heart rate, blood pressure, body temperature, and oxygen saturation. The model is trained on a large dataset consisting of real-world health data, and the results show that the CNN-based approach achieves high accuracy in analyzing and predicting health factors. The authors also discussed the advantages of using CNNs in this context, including their ability to capture complex patterns and relationships in the data. They further highlighted the potential applications of their proposed model, such as remote health monitoring and early detection of health abnormalities. Their article presented a CNN-based health model that demonstrates promising results in analyzing regular health factors in an IoMT environment, paving the way for more advanced and personalized healthcare solutions.

Moreover, Alhussein and Muhammad (2018) presented a mobile multimedia healthcare framework incorporating a voice pathology detection system using DL, specifically CNN models. The system achieved a high accuracy of 98.77% on the SVD database, outperforming previous results. The authors suggested future research directions, including applying parallel CNN models to band-limited voice signals and utilizing a fusion strategy for deep-learned features. Additionally, they proposed combining different types of inputs, such as voice and EGG signals, through a deep fusion strategy. Table 3 discusses the CNN methods used in DL-based methods in medical and health prediction systems.

**Table 3:** Methods, properties, and features of CNN-health prediction systems.

Author	Main idea	Advantage	Disadvantage	Simulation environment	Datasets
Jena et al. (2022)	Proposing a novel 2-branch CNN architecture specifically designed for diabetic retinopathy classification	High accuracy High precision High AUC High specificity High sensitivity	Poor adoptability Poor scalability	–	Images of 102 diabetic patients
Azimi et al. (2018)	Proposing a hierarchical edge computing architecture that leverages DL models at different levels	High accuracy High availability	Poor flexibility	Python	3,600 ECG samples
Lv et al. (2022)	Presenting a novel approach for smart healthcare using DL-based predictive evaluation	High accuracy High scalability High real-time analysis	Poor adoptability	MATLAB	120,000 medical examination data
Ismail et al. (2020)	Presenting a novel approach for analyzing regular health factors in an Internet-of-Medical Things (IoMT) environment using CNNs	High accuracy High f-score High precision High recall	Poor adoptability Poor scalability	MATLAB	Real-time examination of 10,806 patients
Alhussein and Muhammad (2018)	Presenting a mobile multimedia healthcare framework incorporating a voice pathology detection system using DL, specifically CNN models	high accuracy low computational load	Poor scalability	–	4,759,777 records

In summary, showcases advancements in healthcare technology have been discussed comprehensively. Jena et al. developed a specialized CNN for diabetic retinopathy integrated into a smart healthcare system. Azimi et al. propose a hierarchical edge-based DL system for real-time healthcare IoT, tackling bandwidth and privacy issues. Lv et al. introduced a DL-based predictive evaluation for personalized smart healthcare, and Ismail et al. achieved high accuracy in analyzing health factors in an IoMT environment. Additionally, Alhussein and Muhammad present a mobile multimedia healthcare framework with a voice pathology detection system, achieving high accuracy and offering suggestions for future research directions.

### 5.2. Recurrent neural network algorithms for biomedical prediction systems

The role of the RNN method in this context is to effectively capture sequential dependencies and analyze time-series data, enabling tasks such as disease progression modeling, patient monitoring, and prediction of biological processes in healthcare applications. In this regard, Wang et al. (2018) proposed a novel approach for dynamic treatment recommendation using a combination of supervised learning and reinforcement learning techniques. The authors addressed the challenge of making sequential decisions in the context of healthcare, where treatment recommendations may need to be adjusted over time based on patient responses. They introduced an RNN framework that captures temporal dependencies in patient data and uses supervised learning to train the model on historical treatment data. They then integrated reinforcement learning to optimize treatment decisions based on long-term patient outcomes. Their paper presented experimental results that demonstrate the effectiveness of the proposed approach compared to baseline methods, showing improved treatment recommendation accuracy and patient outcomes. The approach held promise for personalized treatment recommendations in healthcare settings, where the ability to adapt treatment strategies based on patient responses is crucial for optimizing patient care.

In addition, Saleem and Chishti (2021) explored the potential advantages and applications of DL in the context of the IoT. Their article discussed various use cases where DL can be applied in IoT, such as smart homes, healthcare monitoring, transportation, and agriculture. It examined the benefits of DL in terms of improved accuracy, automation, adaptability, and scalability in IoT systems. The authors also addressed the challenges and limitations associated with DL in IoT, including data privacy, security, computational complexity, and energy efficiency. Their article additionally presented a model based on LSTM for human activity recognition (HAR) and conducted a comparative analysis of its performance against other ML methods.

Moreover, Jagannatha and Yu (2016) introduced a bidirectional RNN approach for medical event detection in electronic health records (EHRs). The proposed model leveraged the sequential nature of EHR data to capture temporal dependencies and extract meaningful patterns. Through extensive experiments on real-world EHR datasets, the bidirectional RNN demonstrated improved performance in identifying and classifying medical events compared to traditional methods. The study highlighted the potential of utilizing RNNs for medical event detection in EHRs, contributing to the advancement of clinical decision support systems and improving patient care.

In addition, Dami and Yahaghizadeh (2021) focused on the prediction of cardiovascular events using a DL approach in the context of the IoT. The study aimed to develop a predictive model that can effectively identify individuals at risk of cardiovascular events, such as heart attacks or strokes. The authors employed DL techniques, specifically RNN and LSTM networks, to analyze data collected from IoT devices, such as wearable sensors and health monitoring systems. They investigated the impact of different input features, model architectures, and hyperparameters on the performance of the predictive model. The results demonstrated the potential of DL in predicting cardiovascular events, highlighting its applicability in improving risk assessment and early intervention strategies in the IoT-enabled healthcare ecosystem.

In addition, Cocos et al. (2017) explored the application of DL, specifically RNN architectures, for labeling adverse drug reactions (ADRs) in Twitter posts as part of pharmacovigilance efforts. By leveraging the power of RNNs, the study aimed to automatically identify and classify ADRs from the vast amount of user-generated content on social media. Through extensive experiments and evaluation, the proposed RNN models demonstrated promising results in accurately detecting and categorizing ADRs in Twitter posts. The research highlights the potential of DL techniques in pharmacovigilance, offering a valuable approach for monitoring and identifying adverse drug reactions using social media data. Table 4 discusses the RNN methods used in DL-based methods in medical and health prediction systems.

**Table 4:** Methods, properties, and features of RNN health prediction systems.

Author	Main idea	Advantage	Disadvantage	Simulation environment	Dataset
Wang et al. (2018)	Proposing a novel approach for dynamic treatment recommendation	High accuracy	Poor scalability	–	Hospital admission of 43 k patients
Saleem and Chishti (2021)	Presenting a model based on LSTM for Human HAR	High accuracy	Poor adoptability	Python/Keras/ Tensorflow	36 subjects
Jagannatha and Yu (2016)	Introducing a bidirectional RNN approach for medical event detection in electronic health records (EHRs)	High accuracy High recall High precision High F-score	Poor scalability	Lasagne2	99,700 electronic health record notes
Dami and Yahaghizadeh (2021)	Developing a predictive model that can effectively identify individuals at risk of cardiovascular events	Higher accuracy Higher precision Higher F-score	Lack of standards for experimentation and evaluation	MATLAB	9,000 samples
Cocos et al. (2017)	Aiming to automatically identify and classify ADRs from the vast amount of user-generated content on social media	High accuracy High scalability	Poor adoptability Limited size and scope of the dataset	Keras	634-tweet training set

All in all, the text discusses applications of RNNs in healthcare. Wang et al. proposed an RNN-based dynamic treatment recommendation with improved accuracy and patient outcomes. Saleem and Chishti explored DL benefits in IoT, emphasizing accuracy and adaptability, while also addressing challenges. Jagannatha and Yu introduced a bidirectional RNN model for improved medical event detection in EHRs. Dami and Yahaghizadeh focused on early prediction of cardiovascular events in the IoT-enabled healthcare system using DL. Additionally, Cocos et al. effectively used RNNs for labeling adverse drug reactions in Twitter posts, aiding in pharmacovigilance efforts.

### 5.3. Generative adversarial network algorithms for biomedical prediction systems

The role of the GAN method in the trends of using DL algorithms in biomedical prediction systems is to generate synthetic data that closely resemble real biomedical data, allowing for increased data diversity, augmentation, and training of predictive models, ultimately improving the accuracy and generalization capabilities of biomedical prediction systems. Abbood et al. (2022) focused on the development of a GAN for synthesizing diabetic retinopathy (DR) lesions. The authors highlighted the importance of accurate and diverse synthetic data in training computer vision models for DR diagnosis and treatment. They proposed a novel GAN architecture called DR-LL GAN, which consists of a generator network that synthesizes DR lesions and a discriminator network that distinguishes between real and synthetic images. Their article discussed the data preprocessing steps and augmentation techniques used to enhance the quality and diversity of the synthesized DR lesions. The authors evaluated the performance of DR-LL GAN using various evaluation metrics and compared it with other state-of-the-art GAN models. The results demonstrated that DR-LL GAN effectively generates realistic DR lesions, thereby providing a valuable tool for augmenting limited real-world datasets and improving the accuracy of DR detection and diagnosis systems. Their article concluded by discussing the potential applications and future research directions for synthetic DR lesions in the field of computer-aided diagnosis and treatment.

Moreover, Ting et al. (2017) presented the development and validation of a DL system for the detection of diabetic retinopathy (DR) and related eye diseases using retinal images from diverse ethnic populations with diabetes. The authors highlighted the importance of accurate and accessible screening tools for early detection and management of DR. They described the development process of the DL system, which involved training a CNN using a large dataset of retinal images annotated by ophthalmologists. Their article discussed the performance evaluation of the DL system using various evaluation metrics and compared it with human experts and other existing DR detection methods. The results showed that the DL system achieved high accuracy and sensitivity in detecting DR and related eye diseases across different ethnic populations. Their article concluded by emphasizing the potential of the developed DL system as a cost-effective and scalable solution for improving the screening and management of DR in diverse populations with diabetes.

In addition, Waheed et al. (2020) focused on the development of a data augmentation technique using an auxiliary classifier generative adversarial network (AC-GAN) to enhance COVID-19 detection. The authors addressed the challenges posed by limited COVID-19 imaging data, which hinder the performance of DL models in accurately detecting the virus. They proposed CovidGAN, a novel AC-GAN architecture that generates synthetic COVID-19 chest X-ray images while preserving the underlying characteristics and patterns. Their article discussed the training process of CovidGAN, which involves training the generator network to produce synthetic images and the discriminator network to distinguish between real and synthetic images. The authors evaluated the performance of the augmented dataset on different DL models for COVID-19 detection, demonstrating significant improvement in accuracy and sensitivity compared to models trained solely on the original dataset. Their article concluded by highlighting the potential of CovidGAN as a valuable tool for augmenting limited COVID-19 imaging data, ultimately aiding in more accurate and reliable COVID-19 detection systems.

Kadri et al. (2022) presented a novel approach for accurately predicting the length of stay of patients at the emergency department using a DL framework driven by GANs. The authors addressed the challenge of effectively managing resources and providing timely care in emergency departments by developing a predictive model that can estimate the time patients are likely to spend in the emergency department. They proposed a framework that combines a GAN for generating synthetic patient data and a DL model for predicting the length of stay. Their article discussed the training process, where the GAN is trained to generate realistic patient data, and the DL model is trained using the augmented dataset. The authors evaluated the performance of their framework using real-world emergency department data, demonstrating its superiority in accurately predicting patient length-of-stay compared to traditional methods. Their article concluded by highlighting the potential of their GAN-driven DL framework in assisting emergency department staff in resource allocation and decision-making to improve patient flow and overall efficiency.

Purandhar et al. (2022) focused on the classification of clustered healthcare data using GAN. The authors addressed the challenge of analyzing healthcare data that are organized into clusters, where each cluster represents a distinct group or category. They proposed a novel approach that utilized GANs to generate synthetic data samples within each cluster, which can then be used to train classification models. Their article describes the training process of the GAN, where the generator network generates synthetic samples, and the discriminator network distinguishes between real and synthetic samples. The authors evaluate the performance of their approach using real-world healthcare datasets, demonstrating the effectiveness of GAN-based data augmentation for improving classification accuracy in clustered data analysis. Their article concluded by discussing the potential applications of their method in various healthcare domains, such as disease classification, patient risk assessment, and treatment effectiveness evaluation, highlighting the importance of accurate classification in improving healthcare outcomes. Table 5 discusses the GAN methods used in DL-based methods in medical and health prediction systems.

**Table 5:** Methods, properties, and features of GAN-health prediction systems.

Author	Main idea	Advantage	Disadvantage	Simulation environment	Datasets
Abbood et al. (2022)	Focusing on the development of a generative adversarial network (GAN) for synthesizing diabetic retinopathy (DR) lesions	High accuracy High dependability High precision	Limited-size dataset Limited longitudinal tests	–	128 images
Ting et al. (2017)	Presenting the development and validation of a DL system for the detection of diabetic retinopathy (DR) and related eye diseases	High accuracy High AUC High specificity High sensitivity	Poor scalability Poor adoptability	Python	494,661 retinal images
Waheed et al. (2020)	Developing a data augmentation technique using an auxiliary classifier generative adversarial network (AC-GAN) to enhance COVID-19 detection	High accuracy High recall High precision	Poor quality of the synthetic CXR images Limited size of dataset	Keras	721 Normal-CXR images
Kadri et al. (2022)	Presenting a novel approach for accurately predicting the length of stay of patients	High accuracy	Overlooking several parameters for evaluating the method	–	44,676 patients
Purandhar et al. (2022)	Proposing a novel approach that utilizes GANs to generate synthetic data samples within each cluster	High accuracy High specificity	Poor adoptability	–	32 instances

All in all, the text explores GAN applications in biomedical prediction. Abbood et al. synthesized diabetic retinopathy lesions for improved diagnosis. Ting et al. developed a DL system for early detection of eye diseases. Waheed et al. introduced CovidGAN for generating synthetic COVID-19 chest X-ray images. Kadri et al. predicted patient length of stay in emergency departments with a GAN-driven DL framework. Purandhar et al. used GANs for accurate classification in clustered healthcare data analysis.

### 5.4. Long short-term memory algorithms for biomedical prediction systems

The role of the LSTM-based method in this context is to effectively capture and model temporal dependencies in sequential biomedical data, enabling tasks such as disease progression modeling, time-series analysis, and prediction of patient outcomes in healthcare applications. Nancy et al. (2022) presented a smart healthcare monitoring system that utilizes IoT and cloud technologies for predicting heart disease. The authors emphasized the importance of early detection and timely intervention in preventing heart disease-related complications. They proposed a framework that integrates wearable sensors, mobile devices, and cloud computing to collect and analyze patient data, including vital signs and physiological signals. Their article described the DL techniques employed, specifically CNNs and LSTM networks, for feature extraction and prediction of heart disease. The authors evaluated the performance of their system using real-world patient data and compared it with traditional ML algorithms. The results demonstrated the effectiveness of the proposed approach in accurately predicting heart disease and providing real-time monitoring of patients’ health conditions. Their article highlighted the potential of IoT and cloud-based systems in improving healthcare outcomes, enabling early intervention, and reducing the burden on healthcare providers.

Pham et al. (2017) presented a DL approach for predicting healthcare trajectories using medical records. The objective is to forecast future healthcare outcomes and needs of patients based on their historical medical data. The researchers employed a combination of RNN and LSTM networks to capture temporal dependencies and extract relevant features from the sequential nature of medical records. By training the model on a large dataset of patient records, the DL model learns patterns and associations to make accurate predictions. The study demonstrated the effectiveness of the approach through experiments on real-world data, showing superior performance compared to traditional methods. The proposed model has significant potential in proactive care management, identifying high-risk patients, and optimizing healthcare interventions for improved patient outcomes.

Varadharajan and Nallasamy (2022) introduced P-SCADA, a novel FPGA architecture designed for the efficient prediction of heart arrhythmias using LSTM models in BIoT (Biomedical Internet of Things) applications. The proposed architecture focused on optimizing both area and energy efficiency. It leveraged parallel processing and specialized hardware modules to accelerate LSTM computations, enabling real-time prediction of heart arrhythmias while minimizing resource usage and energy consumption. The authors demonstrated the effectiveness of P-SCADA through experiments and comparisons with existing architectures, highlighting its superior performance in terms of prediction accuracy and efficiency. The P-SCADA architecture offered a promising solution for implementing accurate and energy-efficient heart arrhythmia prediction in BIoT applications.

Awais et al. (2020) presented an IoT framework for healthcare and distance learning during the COVID-19 pandemic, focusing on LSTM-based emotion detection using physiological signals. The objective is to develop a system that can detect and analyze emotions based on physiological signals such as heart rate, electrodermal activity, and facial expressions. The proposed framework leveraged the IoT to collect real-time physiological data from individuals remotely. The data are then processed using LSTM networks, a type of RNN, to model the temporal dependencies and extract emotional features. The model is trained and tested using labeled emotion datasets, and performance metrics such as accuracy, precision, recall, and F1-score are used to evaluate its effectiveness. The results showed promising results in emotion detection, highlighting the potential of the framework in healthcare and distance learning applications, particularly during the COVID-19 pandemic where remote monitoring and emotional wellbeing are crucial.

Dong et al. (2019) focused on named entity recognition (NER) in Chinese electronic medical records (EMRs) using DL techniques. The goal is to develop an effective model that can automatically identify and classify important entities such as disease names, medications, and medical procedures within the EMR text. The researchers proposed a novel approach that combines deep transfer learning with multitask bidirectional LSTM RNN (long short-term memory recurrent neural network). The deep transfer learning technique allowed the model to leverage pre-trained word embeddings to capture semantic information and transfer knowledge from other related tasks. The multitask learning approach simultaneously trained the model on multiple NER tasks, enabling it to learn shared representations and improve overall performance. The model is trained and evaluated on a large dataset of Chinese EMRs, and performance metrics such as precision, recall, and F1-score are used to assess its effectiveness. The results demonstrated that the proposed DL model outperforms traditional methods, achieving high accuracy in NER on Chinese EMRs. The findings have implications for improving information extraction and medical data analysis in healthcare systems that utilize Chinese EMRs. Table 6 discusses the LSTM methods used in DL-based methods in medical and health prediction systems.

**Table 6:** Methods, properties, and features of LSTM health prediction systems.

Author	Main idea	Advantage	Disadvantage	Simulation environment	Datasets
Nancy et al. (2022)	Presenting a smart healthcare monitoring system for predicting heart disease	High accuracy High specificity High sensitivity High F-score	High delay Poor scalability	Tensorflow	100,000 records
Pham et al. (2017)	Presenting a DL approach for predicting healthcare trajectories	High accuracy	Poor adoptability	–	7,191 patients with 53,208 admissions
Varadharajan and Nallasamy (2022)	Introducing P-SCADA, a novel FPGA architecture designed for efficient prediction of heart arrhythmias	Energy-efficient Improved computational efficiency Reduced resource utilization	Poor flexibility	Python	BioCreative II GM corpus
Awais et al. (2020)	Presenting an IoT framework for healthcare and distance learning during the COVID-19 pandemic	High accuracy Low latency High reliability	Poor adoptability	Tensorflow	1,000 samples
Dong et al. (2019)	Proposing a novel approach that combines deep transfer learning with multitask bidirectional long short-term memory recurrent neural network (LSTM RNN)	High accuracy High f-Score	Poor scalability	-	300 samples

In summary, the LSTM method effectively models temporal dependencies in biomedical data. Nancy et al. employed IoT and cloud tech for heart disease prediction, emphasizing early detection. Pham et al. used DL to forecast patient healthcare trajectories, outperforming traditional methods. Varadharajan and Nallasamy introduced P-SCADA, an FPGA architecture for efficient heart arrhythmia prediction. Awais et al. presented an IoT framework for remote emotion detection using LSTM. Dong et al. applied DL for named entity recognition in Chinese EMRs, achieving high accuracy.

### 5.5. Support vector machine algorithms for biomedical prediction systems

The role of the SVM method in this context is to provide a robust and interpretable classification framework, complementing DL models by offering a different approach to feature extraction and classification in biomedical data analysis. Ahmad et al. (2020) focused on the development of accurate models for short-term electricity load forecasting. The authors addressed the importance of accurate load forecasting in electricity grid management and proposed an improved approach using SVM and extreme learning machines (ELMs). They described the enhancements made to traditional SVM and ELM algorithms, such as parameter optimization and feature selection, to improve the forecasting accuracy. Their article discussed the experimental setup and evaluation of the proposed models using real-world electricity load data. The results demonstrated that the improved SVM and ELM models outperform the baseline models and achieve higher accuracy in short-term load forecasting. Their article concluded by highlighting the potential of the proposed models in assisting power system operators and electricity market participants in making informed decisions and improving the efficiency and reliability of the electricity grid.

Muhammad et al. (2021) explored the use of supervised ML models to predict COVID-19 infection based on epidemiology datasets. The authors highlighted the importance of accurate prediction models in managing and controlling the COVID-19 pandemic. They investigated various ML algorithms, including logistic regression, decision trees, random forests, SVM, and ANNs, to forecast the likelihood of COVID-19 infection using a wide range of epidemiological variables. To train and evaluate their models, the authors utilized a comprehensive dataset comprising demographic, clinical, and environmental factors. They conducted experiments and assessed model performance using metrics such as accuracy, precision, recall, and F1-score. The findings revealed the effectiveness of supervised ML models in accurately predicting COVID-19 infection based on the provided dataset. This research has significant implications for early detection, risk assessment, and resource allocation during the ongoing COVID-19 pandemic.

Cohen et al. (2016) addressed the challenges and considerations associated with predicting candidates for pediatric epilepsy surgery using NLP and ML techniques. The authors discussed the importance of accurate identification and selection of suitable patients for epilepsy surgery to improve treatment outcomes. They described the process of developing a prediction model using NLP algorithms to extract relevant features from clinical narratives and ML algorithms to classify patients as surgery candidates or non-candidates. Their article highlighted several methodological issues, including data preprocessing, feature selection, model training, and evaluation. The authors also discussed potential limitations, such as the availability and quality of clinical data, the interpretability of ML models, and the generalizability of the results. Their article provided valuable insights into the methodological considerations and challenges involved in using NLP and ML for predicting pediatric epilepsy surgery candidates, offering guidance for future research in this field.

Kaur and Kumari (2022) explored the application of ML techniques in predicting and analyzing diabetes. The authors emphasized the significance of accurate predictions in managing and preventing the disease. They highlighted the limitations of traditional statistical methods and proposed the use of ML algorithms to improve prediction accuracy. The study discussed various ML algorithms, such as decision trees, SVMs, and neural networks, and their suitability for diabetes prediction. The authors also addressed the importance of feature selection and data preprocessing techniques in enhancing the performance of these algorithms. They concluded that ML approaches offer promising avenues for predicting and analyzing diabetes, providing valuable insights for healthcare professionals and patients in the prevention and management of the disease.

Amer et al. (2022) presented a novel system called BioLearner, which utilizes ML techniques to predict the risk of heart disease based on biomedical markers. The authors emphasized the importance of early detection and prevention of heart disease as it is a leading cause of mortality worldwide. They discussed the limitations of traditional risk assessment models and proposed the integration of ML algorithms to enhance prediction accuracy. The authors described the development of BioLearner, which incorporates various ML algorithms, including decision trees, random forests, and gradient boosting, to analyze a wide range of biomedical markers such as cholesterol levels, blood pressure, and genetic information. They validated the performance of BioLearner using real-world datasets and compared it with existing risk prediction models, demonstrating its superior accuracy and effectiveness. Their article concluded that BioLearner has the potential to serve as a smart system for heart disease risk prediction, enabling early intervention and personalized healthcare decisions. Table 7 discusses the SVM methods used in DL-based methods in medical and health prediction systems.

**Table 7:** Methods, properties, and features of SVM health prediction systems.

Author	Main idea	Advantage	Disadvantage	Simulation environment	Datasets
Ahmad et al. (2020)	Developing accurate models for short-term electricity load forecasting	High accuracy	Poor scalability	Python	The daily electricity load data of 3 years from Independent System Operator New England
Muhammad et al. (2021)	Exploring the use of supervised ML models to predict COVID-19 infection	High accuracy High specificity High sensitivity	Poor adoptability	Python	475 viral respiratory disease monitoring units
Cohen et al. (2016)	Developing a prediction model using NLP algorithms to extract relevant features from clinical narratives and ML algorithms	Low Lag time High accuracy	Poor flexibility	–	A sample of 62 patients
Kaur and Kumari (2022)	Exploring the application of ML techniques in predicting and analyzing diabetes	High accuracy High F-score High precision High AUC High recall	Poor adoptability	R	768 instances
Amer et al. (2022)	Presenting a novel system called BioLearner to predict the risk of heart disease	High accuracy	Poor adoptability	Python/Keras	165 records

In brief, their article highlights various applications of SVM and ML techniques in biomedical data analysis. Studies by Ahmad, Muhammad, Cohen, Kaur, and Amer demonstrate the effectiveness of these approaches in improving accuracy and early detection in fields such as electricity load forecasting, COVID-19 prediction, epilepsy surgery candidacy, diabetes management, and heart disease risk assessment.

### 5.6. Hybrid algorithms for biomedical prediction systems

The role of hybrid methods in this regard is to leverage the complementary strengths of different models or techniques, combining DL with traditional ML or statistical approaches, to enhance performance, interpretability, and robustness in biomedical prediction tasks. Umer et al. (2022) focused on the development of an Internet-of-Things (IoT)-based smart monitoring system for patients with acute heart failure. The objective was to create a remote monitoring solution that can continuously collect and analyze vital signs and other relevant data from patients, enabling early detection of heart failure symptoms and timely interventions. The proposed system utilized various IoT devices, including wearable sensors and mobile applications, to gather real-time information such as heart rate, blood pressure, oxygen saturation, and activity levels. The collected data are then transmitted to a central server where it is processed and analyzed using ML algorithms. The system can detect anomalies or changes in the patient’s vital signs that may indicate the onset of acute heart failure. Alerts are sent to healthcare providers or caregivers, enabling prompt action to be taken. Their article discussed the design and implementation of the IoT-based monitoring system, as well as its potential benefits in terms of improving patient outcomes, reducing hospital readmissions, and enabling remote patient management for individuals with acute heart failure.

Yamamoto et al. (2020) introduced a hybrid DL model that combines CNNs and LSTM networks for the reconstruction of electrocardiogram (ECG) signals using a Doppler sensor. The goal is to develop a method for accurately capturing ECG signals using a non-contact sensing approach, which can be beneficial in scenarios where traditional ECG electrodes are not feasible or comfortable for patients. The proposed model leveraged the CNN to extract spatial features from the Doppler signals, while the LSTM network captures the temporal dependencies in the reconstructed ECG waveform. The model is trained on a dataset of synchronized Doppler and ECG recordings and evaluated using performance metrics such as mean squared error (MSE) and peak signal-to-noise ratio (PSNR). The results demonstrated the effectiveness of the hybrid DL model in accurately reconstructing ECG signals from Doppler sensor data, offering potential applications in remote monitoring and non-invasive cardiac diagnostics.

Ketu and Mishra (2022) provided an Indian perspective on utilizing a CNN-LSTM hybrid DL model for COVID-19 prediction and assessing the current status of medical resource availability. The study aimed to develop a predictive model that can forecast COVID-19 cases and analyze the availability of medical resources in India. The CNN component of the model is designed to extract spatial features from input data, while the LSTM component captures temporal dependencies. The model is trained on a dataset of COVID-19 cases and medical resource data specific to India. It is evaluated using performance metrics such as accuracy, precision, recall, and F1-score. The results demonstrated the effectiveness of the model in predicting COVID-19 cases and providing insights into the availability of medical resources. This information can assist healthcare authorities and policymakers in making informed decisions regarding resource allocation and management during the pandemic in India.

Munagala et al. (2022) presented a smart IoT-enabled heart disease monitoring system that utilizes a meta-heuristic-based fuzzy LSTM model. The objective is to develop an intelligent system that can accurately monitor and predict heart disease using a combination of IoT devices and advanced ML techniques. The proposed system integrated IoT devices such as wearable sensors and mobile applications to collect real-time physiological data from individuals. The data are then processed and analyzed using a fuzzy-LSTM model, which combines fuzzy logic with LSTM networks. The fuzzy logic component allowed for handling uncertainties and imprecise data, while the LSTM network captured the temporal dependencies in the data. The model is trained and evaluated using heart disease datasets, and performance metrics such as accuracy, sensitivity, specificity, and F1-score are utilized. The results demonstrated the effectiveness of the proposed system in heart disease monitoring and prediction, offering potential benefits in early detection and proactive management of heart conditions.

Garcia-Moreno et al. (2020) presented a CNN-LSTM DL classifier for detecting motor imagery EEG (electroencephalogram) signals using a low-invasive and low-cost BCI (brain-computer interface) headband. The goal is to develop a system that can accurately recognize specific mental commands related to motor activities from EEG signals, enabling control of external devices through brain signals. The proposed system utilized a combination of CNN and LSTM networks. The CNN component extracted spatial features from the EEG signals, while the LSTM network captured the temporal dependencies. The classifier is trained and tested using a dataset of motor imagery EEG recordings obtained through the low-invasive headband. Performance evaluation metrics such as accuracy, precision, recall, and F1-score are utilized. The results demonstrated the effectiveness of the CNN-LSTM classifier in accurately detecting motor imagery EEG signals, showcasing the potential of the low-cost BCI headband for practical and accessible brain-controlled applications. Table 8 discusses the hybrid methods used in DL-based methods in medical and health prediction systems.

**Table 8:** Methods, properties, and features of SVM health prediction systems.

Author	Main idea	Advantage	Disadvantage	Simulation environment	Datasets
Umer et al. (2022)	Developing an Internet of Things (IoT) based smart monitoring system for patients with acute heart failure	High accuracy High AUC High F-score High precision	Poor flexibility	Tensorflow	299 patient records
Yamamoto et al. (2020)	Introducing a hybrid DL model that combines CNNs	High accuracy	Poor accuracy of ECG signal reconstruction Poor robustness	–	24 GHz Doppler sensor with the sampling rate of 1,000 Hz
Ketu and Mishra (2022)	Providing an India perspective on utilizing a CNN-LSTM hybrid DL model for COVID-19 prediction	Minimal MAPE R2 Score Minimal RMSE	Poor scalability	Python	Adaptive moment estimation (ADAM) with a batch size of 64
Munagala et al. (2022)	Presenting a smart IoT-enabled heart disease monitoring system	High accuracy High precision	Limited size of dataset Poor scalability	Python	14,552 samples
Garcia-Moreno et al. (2020)	Presenting a CNN-LSTM DL classifier for detecting motor imagery EEG	High accuracy	Limited sample size Poor integrity Awkward to wear in outdoor activities	Python/ Keras	Four healthy users aged from 33 to 55

## 6. Results and comparisons

The utilization of DL-based methods in medical and health prediction systems purposes represents a pioneering stride toward the progress of the medical and healthcare industries. This article presents various innovative applications that demonstrate this paradigm, showcasing advanced knowledge in image segmentation to motivate readers to explore innovative categories pertaining to medical-ML applications in healthcare. The primary focus of this work is on different classifications of ML techniques utilized for medical and health prediction systems. Through a comprehensive analysis, it has been discovered that most DL-based methods in medical and health prediction systems concentrate on advanced datasets, combined learning tasks, and annotation protocols. However, a significant limitation toward achieving the same level of functionality in healthcare-ML applications is the inadequacy of large datasets for training and standardized collection of data. It is crucial to ensure that diverse types of data require larger and more diverse datasets to provide reliable outcomes. Detection tasks in this field predominantly employ CNN or CNN-based techniques. In most of the investigated articles, the authors evaluated the topic based on several attributes, including accuracy, F-score, AUC, sensitivity, specificity, robustness, recall, adaptability, and flexibility. Sections 5.1–5.5 illustrate the healthcare-ML application in medical and healthcare prediction systems, where the majority of the proposed methods use both benchmark and real-time data. The systems employed various datasets in terms of numbers and diverse categories, with accuracy, AUC, sensitivity, specificity, robustness, flexibility, adaptability, scalability, and F-score being the primary parameters evaluated. Accuracy was the main parameter for DL-based methods in medical and health prediction systems, whereas integrity was the least applied parameter. However, since different datasets were used in the research, some of the provided systems did not demonstrate the computing time. The datasets utilized in the study had different features, including the number of samples, accessibility conditions, image size, and classes. CNN is commonly used in this context due to its effectiveness in processing and analyzing complex visual and spatial data. CNNs excel at extracting meaningful features from images, which is crucial in biomedical applications such as medical image analysis and pathology detection. They have demonstrated high accuracy in tasks such as image segmentation, classification, and anomaly detection, making them a popular choice for researchers in the field. Additionally, CNNs can handle large amounts of data, learn hierarchical representations, and adapt to variations and complexities within biomedical datasets, further contributing to their widespread adoption in this domain.

**Figure 8:**
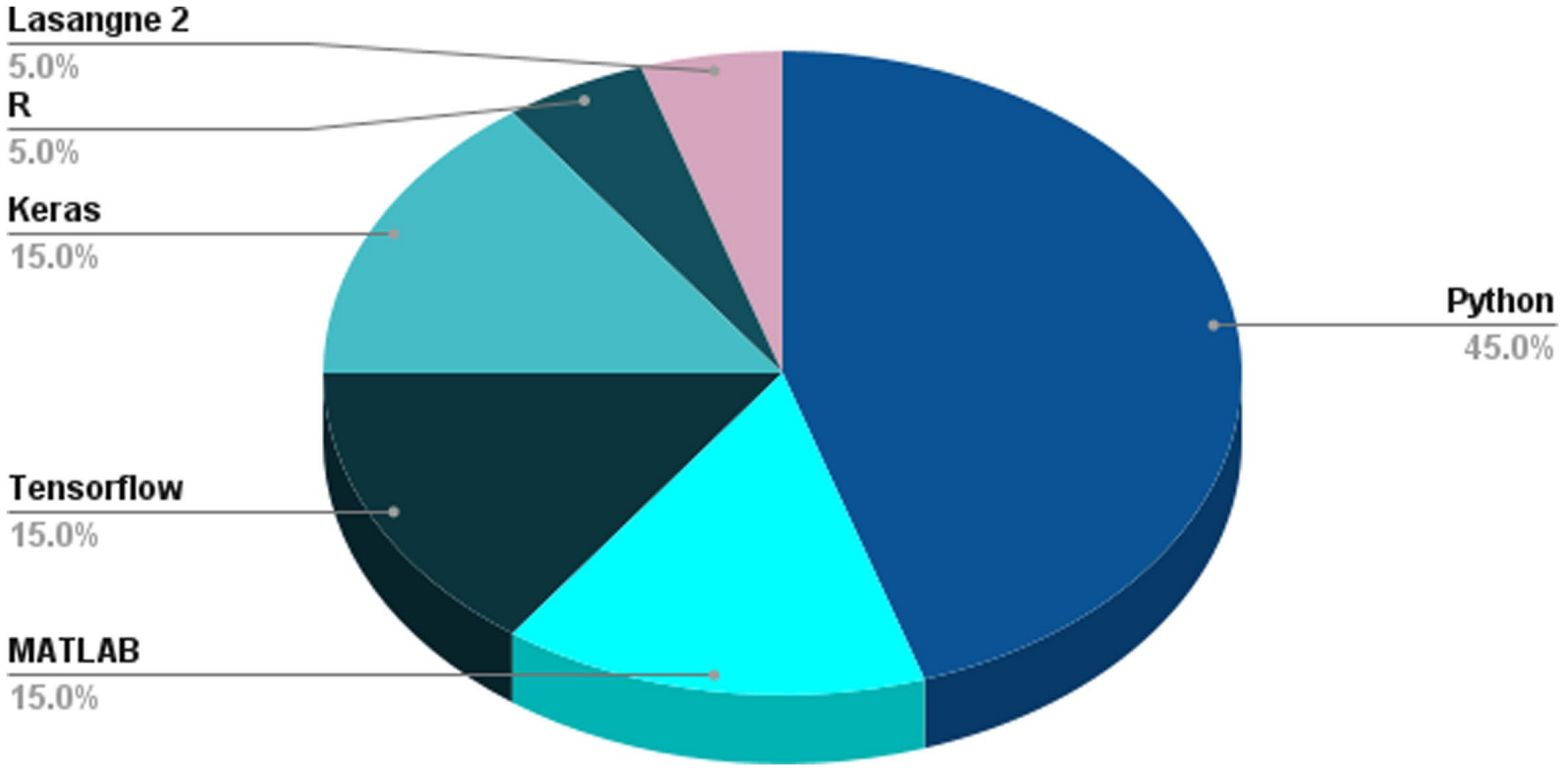
DL Methods used in medical and health prediction systems.

**Figure 9:**
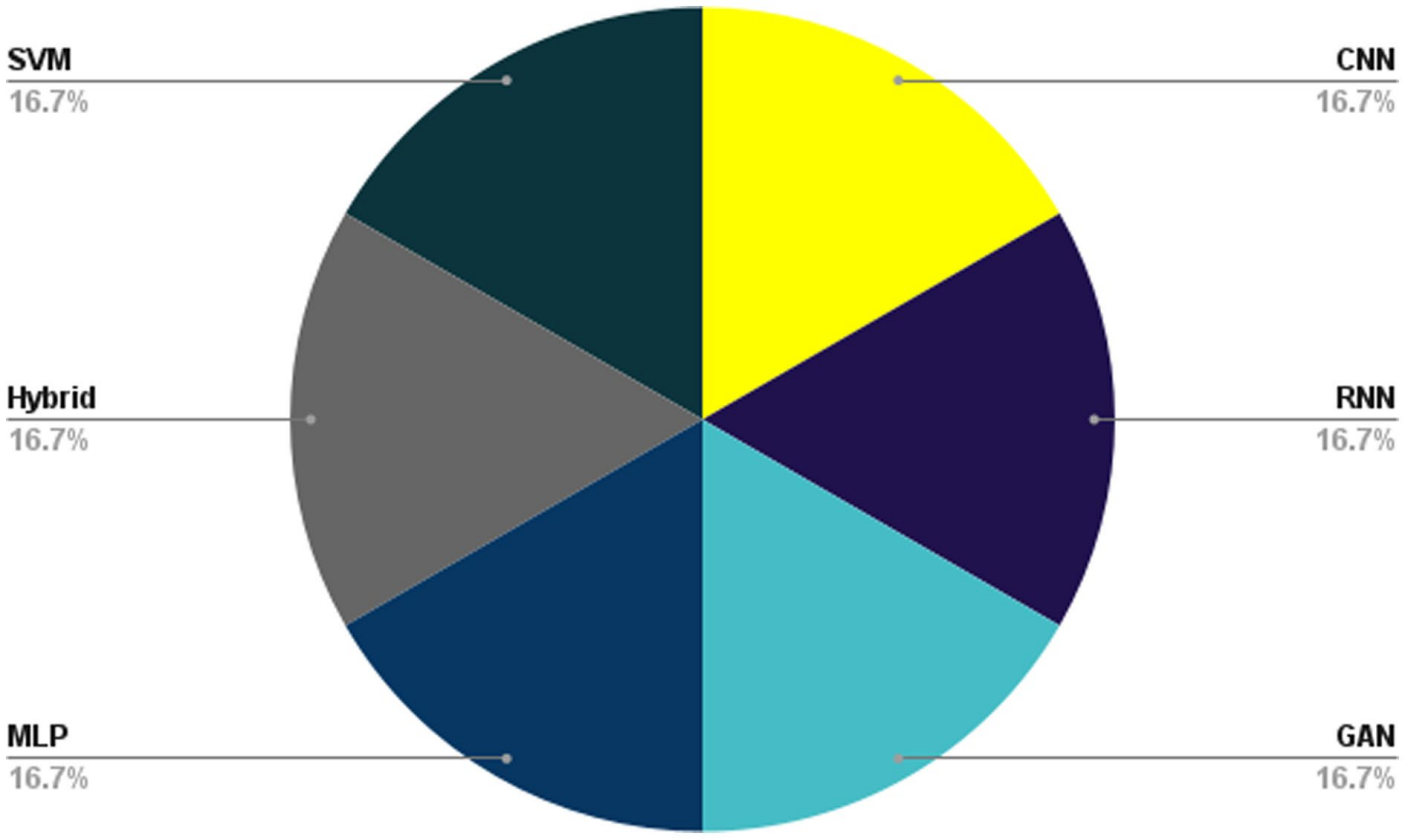
Programming languages used in DL-medical and health prediction systems.

**Figure 10:**
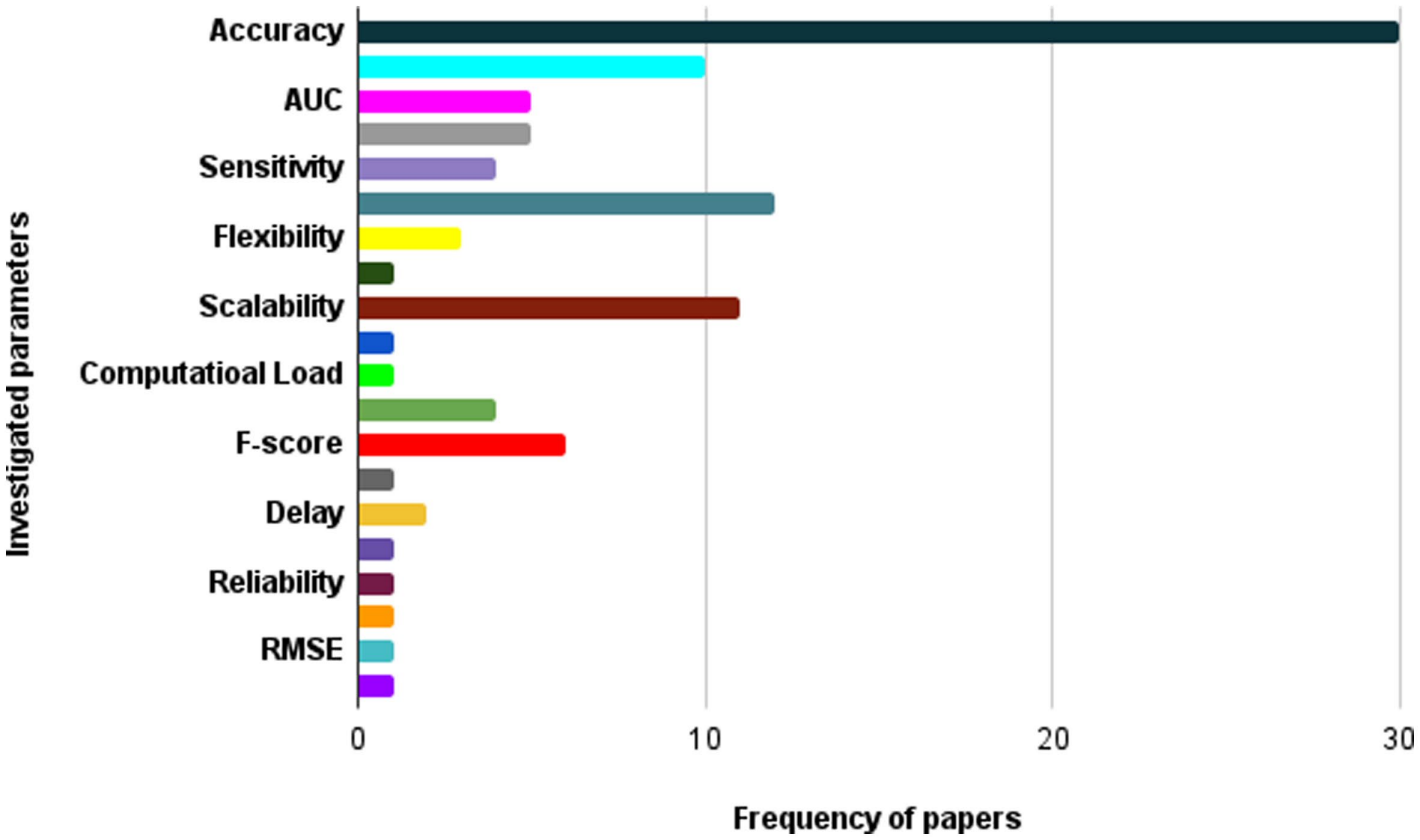
Parameters used in deep symmetry application in DL-medical and health prediction systems.

In the preceding sections, we scrutinized a corpus of 30 articles encompassing six categories that pertain to the implementation of DL techniques in medical and health prediction systems, as depicted in Figure 8. Notably, Python emerged as the most frequently utilized language in simulating, implementing, or establishing theoretical frameworks, thus rendering it highly practical for researchers to employ in forthcoming research, owing to its extensive domain of applicability as shown in Figure 9. Using Python in these studies is due to several reasons. First, Python has a rich ecosystem of libraries and frameworks specifically designed for ML and DL, such as TensorFlow, PyTorch, and Keras. These libraries provide extensive functionality and easy-to-use interfaces for implementing and training DL models. Second, Python is known for its simplicity and readability, which makes it more accessible for researchers and developers in the field of biomedical prediction systems. It allows them to express complex ideas and algorithms in a concise and understandable manner. Additionally, Python has a large and active community, providing abundant resources, tutorials, and support for DL applications. Finally, Python’s versatility and compatibility with other scientific computing libraries enable seamless integration with data processing, visualization, and analysis tools commonly used in biomedical research. Overall, these factors contribute to the widespread adoption of Python in the field of DL for biomedical prediction systems. The choice of framework ultimately depends on factors such as personal preference, existing expertise, and specific requirements of the project. Moreover, as Figure 10 depicts, accuracy is the most important parameter considered in investigated articles.

### 6.1. Convolutional neural network

**Figure 11:**
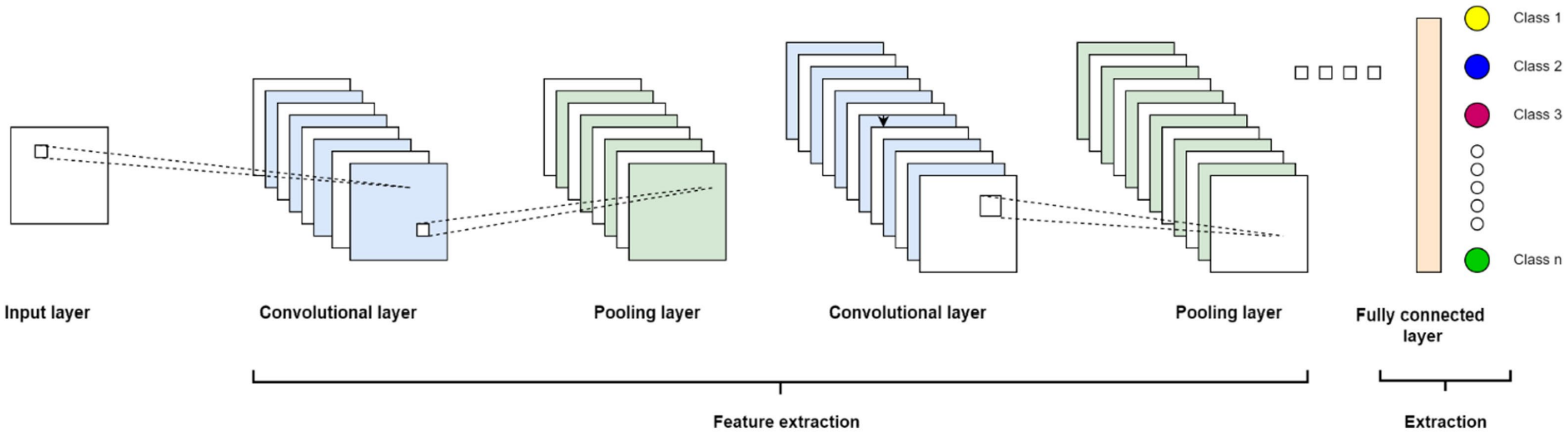
Architecture of CNN framework.

A CNN, as shown in Figure 11, is composed of a stack of three primary neural layers, including the convolutional layer, the pooling layer, and the fully connected layer which perform feature extraction by applying filters to the input image. Each layer performs a distinct function. These layers are typically followed by pooling layers that downsample the feature maps, reducing their spatial dimensions. Subsequently, fully connected layers are used for classification or segmentation, integrating the extracted features for final prediction (Gao et al., 2022). This hierarchical structure of CNNs allows them to capture spatial relationships and patterns in medical images, facilitating accurate segmentation of regions of interest. The convolution layer detects unique features, such as edges or other visual elements, in an image. This layer carries out a mathematical operation involving the multiplication of local neighbors of an image pixel with kernels. To generate its feature maps, CNN uses different kernels for convolving the given image. The pooling layer reduces the spatial dimensions (width, height) of the input data for the subsequent neural network layers, without altering its depth. This process is known as subsampling. The size reduction of the pooling layer decreases the computational requirements for the upcoming layers (Jin et al., 2023). The fully connected layers perform high-level reasoning in NN, integrating the various feature responses from the given input image to provide the final results.

There are several advantages of using CNN in the studied articles. In the diabetic retinopathy classification article, CNNs excel at analyzing retinal images, capturing complex features and patterns that contribute to accurate classification (Chen et al., 2017). In the healthcare IoT system article, CNNs efficiently process image and video data at the network edge, reducing latency and conserving bandwidth. In the smart predictive evaluation article, CNNs extract visual features from multimedia data, such as images and videos, enabling accurate prediction in interactive healthcare scenarios (Asghari et al., 2018). In the health factors analysis article, CNNs are adept at analyzing medical images or sensor data, facilitating the analysis of regular health factors in an IoMT environment. In the medical big data analysis article, CNNs handle the complexities of visual data, enabling the extraction of valuable insights from large-scale datasets in IoT-based medical data analysis (Shahid and Singh, 2019).

The use of CNN in trends involving DL algorithms in biomedical prediction systems also comes with its own set of challenges. One challenge is the need for a large amount of labeled training data, which may be limited in the biomedical domain due to privacy concerns or the difficulty of obtaining annotated data. Insufficient training data can hinder the ability of CNNs to learn complex patterns and generalize well to unseen data. Another challenge is the interpretability of CNN models (Tufail et al., 2021). CNNs are often regarded as black-box models, making it challenging to understand the features and reasoning behind their predictions, which is crucial in the biomedical field for trust, validation, and explainability. Additionally, CNNs may not be effective for capturing temporal dependencies and sequential patterns in certain biomedical data types, such as time-series or longitudinal data (Srivastava et al., 2017). Handling class imbalance, dealing with noisy or incomplete data, and addressing issues related to overfitting are also challenges that need to be considered when using CNNs in biomedical prediction systems. Addressing these challenges requires careful data preparation, model design, regularization techniques, and evaluation strategies to ensure the reliability and performance of CNN-based models in biomedical applications (Dalal and Khalaf, 2021).

### 6.2. Recurrent neural network

**Figure 12:**
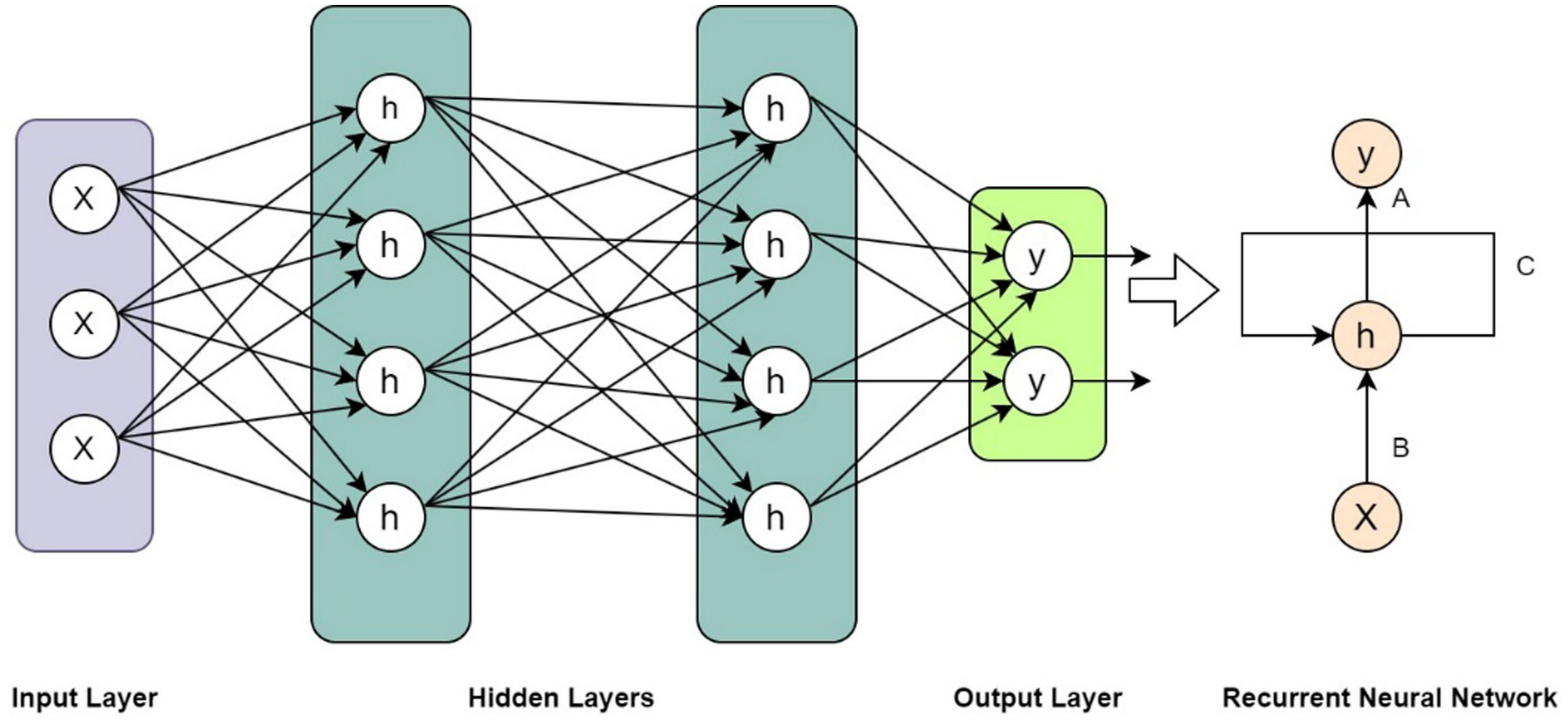
Architecture of RNN framework.

On the other hand, RNNs are commonly used in this research for several reasons. RNNs are well-suited for processing sequential data, which is prevalent in many biomedical applications. They are particularly effective in capturing temporal dependencies and modeling sequences of data. In the context of biomedical prediction systems, RNNs can be used to analyze time-series data, such as physiological signals or patient records, and make predictions or classifications based on the temporal dynamics of the data. RNNs can also handle variable-length input sequences, making them suitable for tasks where the length of the data may vary (Vinitha et al., 2018). Additionally, RNN variants such as LSTM and Gated Recurrent Unit (GRU) address the issue of vanishing gradients, enabling them to capture long-term dependencies more effectively. The ability of RNNs to model sequential data and capture temporal relationships makes them valuable tools in biomedical prediction systems, enabling tasks such as disease diagnosis, patient monitoring, and treatment outcome prediction. Considering Figure 12, the RNN method, utilized in deep symmetry applications for image segmentation, has a distinct structure. It incorporates recurrent connections that enable information to persist across sequential inputs, making it suitable for analyzing images as a sequence of pixels. RNNs consist of recurrent layers, such as LSTM or Gated Recurrent Unit (GRU), which capture dependencies and relationships between pixels. These layers are connected to each other, allowing the network to learn contextual information and long-range dependencies required for accurate segmentation. This memory-based structure of RNNs aids in incorporating global and local information for informed segmentation decisions in medical image analysis.

However, the use of RNNs in this study presents certain challenges. One challenge is the vanishing or exploding gradient problem, which can occur when training RNNs on long sequences. This can lead to difficulties in capturing long-term dependencies and can hinder the model’s ability to learn meaningful patterns. Another challenge is the computational complexity of training RNNs, especially when dealing with large-scale biomedical datasets (Kakhi et al., 2022). The sequential nature of RNNs makes them more computationally intensive compared to other architectures, which can impact training time and resource requirements (Chen et al., 2022). Additionally, RNNs may struggle with modeling very long sequences due to memory limitations and the loss of relevant information from earlier time steps. Another challenge is the proper handling of irregular or missing data in biomedical applications (Shamshirband et al., 2021). RNNs are sensitive to missing values or irregularly sampled data, and special care must be taken to handle these cases appropriately. Finally, the interpretability of RNNs can be a challenge, as the inner workings of the model and the reasoning behind its predictions are not always transparent. Addressing these challenges requires advancements in training techniques, model architectures, handling missing data, and ensuring interpretability to maximize the effectiveness of RNNs in biomedical prediction systems (Panganiban et al., 2019).

### 6.3. Generative adversarial network

**Figure 13:**
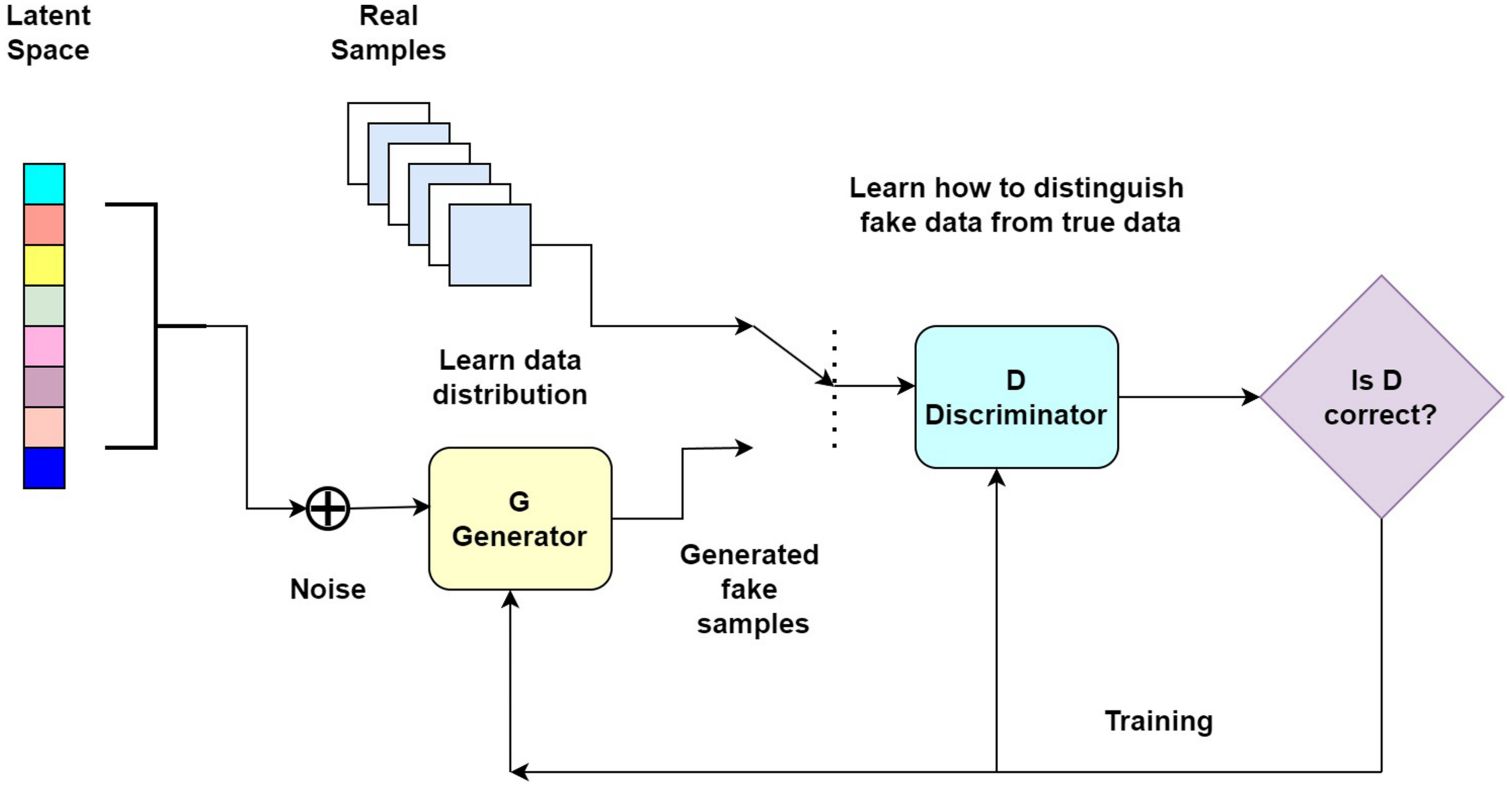
Architecture of GAN framework.

An example of a network that trains two models concurrently *via* unsupervised learning is a GAN. The training of two DL models in tandem is one of the primary objectives of GANs. These networks are made up of the discriminator (D) and generator models (G). The discriminator categorizes a certain model that comes from the training data or the generator, whereas the generator creates fresh samples or instances. Using GAN in the network is a solution when the volume of data is lower than usual. The generator’s goal is to be able to produce a false output that is as close to the genuine output as possible. The generator’s output must be so similar to the genuine one that it is impossible to tell the difference between the real and false data. GANs have three main phases: (1) In the first phase, the generator selects erroneous numbers and outputs a picture. (2) The discriminator takes both actual and false pictures in the second step, returning probabilities in both cases. In this phase, we have two probabilities, 0 and 1. The output from the generator contains bogus data if it is near 0 and actual data if it is close to 1. (3) In the third stage, the generator is trained, and its output is improved using feedback from the discriminator network. To classify brain tumors based on MRI scans, a DNN with GAN pre-training was used. It was used as a discriminator in a GAN architecture to pre-train the deep network, which significantly improved overall performance. The architecture of the GAN framework is shown in Figure 13.

Although GANs are used in this research for their ability to generate synthetic data that closely resembles real biomedical data, GANs consist of a generator network and a discriminator network that work in opposition to each other, where the generator tries to produce realistic synthetic samples and the discriminator aims to differentiate between real and generated samples (Wei et al., 2020; Yin et al., 2022). In the context of biomedical prediction systems, GANs can be employed to generate synthetic data that captures the distribution and characteristics of real biomedical data. This is particularly useful when the available real data is limited or when privacy concerns restrict access to sensitive patient data. GANs can be trained on a large dataset of real data and then used to generate additional synthetic data, which can be used to augment the training set and improve the performance of prediction models. Additionally, GANs can help in data augmentation, anomaly detection, and feature extraction tasks in biomedical prediction systems (Cheng et al., 2016). By leveraging the power of GANs, researchers can enhance the availability and quality of data for training and testing predictive models in the biomedical domain.

The use of GANs in trends involving DL algorithms in biomedical prediction systems is not without its challenges. One challenge is the difficulty in training GANs, as they require a delicate balance between the generator and discriminator networks. GAN training can be unstable, leading to issues such as mode collapse, where the generator fails to capture the full distribution of the data (Aghdam et al., 2021). Another challenge is the generation of high-quality and realistic synthetic data. While GANs can produce synthetic samples, ensuring their fidelity to real biomedical data can be a complex task. Additionally, GANs may suffer from a lack of interpretability, making it challenging to understand the generated data or the reasoning behind the predictions made by GAN-based models. Finally, the performance of GANs can be highly dependent on the availability and quality of the training data. Insufficient or biased training data can impact the effectiveness and generalizability of GAN-based models in biomedical prediction systems. Addressing these challenges is crucial to harness the potential of GANs for improving the accuracy and reliability of predictions in the biomedical domain (Mittal and Hasija, 2020; Zhu et al., 2020).

### 6.4. Long short-term memory

LSTM networks are used in research on this topic for several reasons. One of the main reasons is LSTM’s ability to effectively capture and model long-term dependencies in sequential data, which is often prevalent in biomedical data such as time-series or EHRs. The memory cells in LSTM networks allow them to retain information over longer sequences, making them suitable for tasks where context and temporal relationships are crucial. Additionally, LSTMs can mitigate the vanishing or exploding gradient problem that can occur in traditional RNNs, enabling more stable and effective training. The gated architecture of LSTMs, with their input, output, and forget gates, helps regulate the flow of information and selectively retain or discard relevant information at each time step. This makes LSTMs well-suited for tasks such as time-series forecasting, disease progression prediction, anomaly detection, and other biomedical prediction problems where capturing and modeling temporal dependencies are critical.

The advantage of using the LSTM method in trends of DL algorithms in biomedical prediction systems lies in its ability to effectively capture and model long-term dependencies in sequential data. Biomedical data often exhibit temporal dependencies and LSTM networks excel at capturing these dependencies over extended sequences (Ji et al., 2021). The memory cells in LSTMs enable them to retain and selectively forget information over time, allowing for the preservation of relevant context. This makes LSTMs well-suited for tasks such as time-series forecasting, disease progression prediction, and other biomedical prediction problems where understanding the temporal relationships is crucial. Furthermore, LSTMs address the vanishing or exploding gradient problem encountered in traditional RNNs, ensuring more stable and effective training. Overall, the LSTM method enhances the performance of DL models in biomedical prediction systems by effectively modeling long-term dependencies and handling sequential data (Miotto et al., 2018).

While LSTM networks offer advantages in biomedical prediction systems, they also pose some challenges. One challenge is the increased complexity and computational requirements associated with LSTM models, which can require more time and resources for training and inference compared to simpler models. Another challenge is the potential for overfitting, where the model becomes too specialized to the training data and fails to generalize well to unseen data. Regularization techniques such as dropout and early stopping can help mitigate this issue (Mishra et al., 2022). Additionally, determining the optimal architecture and hyperparameters for LSTM models can be challenging, as the performance may vary based on factors such as the number of layers, hidden units, and sequence lengths. Proper model selection and tuning are important to achieve optimal performance. Finally, interpreting the decisions made by LSTM models can be challenging, as they are often regarded as black-box models. Methods such as attention mechanisms or interpretability techniques can be employed to gain insights into the model’s decision-making process. Overall, addressing these challenges is crucial to effectively utilize LSTM in biomedical prediction systems and ensure accurate and interpretable results (Rafiei et al., 2021).

**Figure 14:**
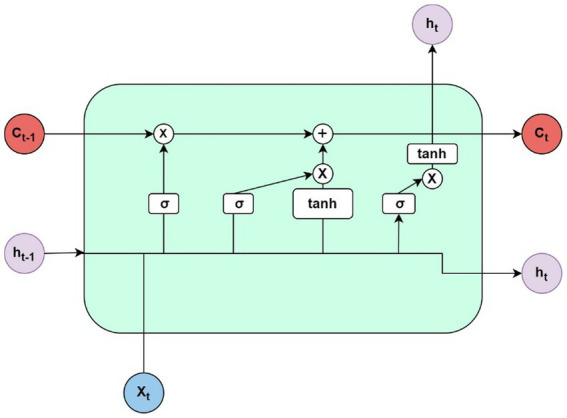
Architecture of LSTM framework.

Figure 14 shows the architecture of the LSTM framework. LSTM networks consist of memory cells that allow for the retention and utilization of information over time. The structure includes gates that regulate the flow of information, such as input, forget, and output gates, enabling effective modeling of long-range dependencies and capturing contextual information. By incorporating LSTM layers, the network can effectively analyze the spatial relationships and symmetrical properties present in medical images, leading to accurate segmentation results and an improved understanding of the underlying structures.

### 6.5. Support vector machine

SVM is used in research on trends in using DL algorithms in biomedical prediction systems for several reasons. First, SVM is a powerful and well-established classification algorithm that can handle both linear and non-linear data. It has been widely applied in various domains, including biomedicine, due to its ability to handle high-dimensional data and handle class imbalance. Second, SVM has a solid theoretical foundation and good generalization properties, making it suitable for biomedical prediction tasks with limited labeled data (Savova et al., 2019). Additionally, SVM allows for the use of different kernel functions, enabling the modeling of complex relationships between features and labels. Finally, SVM provides a probabilistic output through the use of techniques such as Platt scaling, which can be beneficial for decision-making and risk assessment in biomedical applications.

One advantage of using SVM in trends of using DL algorithms in biomedical prediction systems is its ability to handle high-dimensional data. Biomedical data often contains a large number of features, and SVM can effectively handle such complex and high-dimensional datasets. Additionally, SVM has a strong theoretical foundation and good generalization properties, allowing it to perform well even with limited training data. SVM is also known for its ability to handle class imbalance, which is common in biomedical datasets where certain classes may be under-represented (Hamidinekoo et al., 2018). Moreover, SVM provides a probabilistic output, which can be useful for risk assessment and decision-making in biomedical applications.

There are a few challenges associated with using SVM in trends of using DL algorithms in biomedical prediction systems. SVM can be computationally expensive, especially when dealing with large datasets or high-dimensional feature spaces. The training time and memory requirements of SVM increase significantly as the size of the dataset grows, which can limit its scalability in certain applications. SVM has hyperparameters that need to be carefully tuned, such as the choice of kernel function and regularization parameter (Nisha and Meeral, 2021). The performance of SVM can be sensitive to the selection of these parameters, and finding the optimal combination can be a challenging task, requiring extensive experimentation and cross-validation. SVM assumes that the data is linearly separable or can be transformed into a separable space using the kernel trick. However, in biomedical prediction systems, the data may be noisy or contain overlapping classes, which can make it difficult for SVM to find an optimal decision boundary. SVM’s memory requirements can be a limiting factor when dealing with large-scale biomedical datasets. In scenarios where the dataset size exceeds the available memory, SVM may not be feasible or require extensive data preprocessing or dimensionality reduction techniques. Despite these challenges, SVM remains a widely used and effective ML algorithm in biomedical prediction systems (Nogales et al., 2021). Careful consideration of the dataset characteristics and appropriate parameter tuning can help mitigate these challenges and improve the performance of SVM in this context.

Hybrid methods in biomedical prediction systems aim to enhance performance by combining the strengths of DL algorithms, such as CNNs and RNNs, with traditional ML techniques such as SVM and decision trees. DL excels in learning complex patterns from large datasets but may struggle with small datasets or temporal dependencies. Hybrid models leverage the feature extraction expertise of DL and the interpretability of traditional ML, allowing for better handling of data scarcity and imbalanced classes (Ye et al., 2022). They also enable the incorporation of domain knowledge, enhancing interpretability. However, hybrid models can introduce complexity, require additional computational resources, and pose challenges in model selection and hyperparameter tuning. Interpretability may also be compromised in complex hybrid architectures. Careful consideration of these factors is essential when implementing hybrid methods in biomedical prediction systems.

### 6.6. Prevalent evaluation criteria

In the trends of using DL algorithms in biomedical prediction systems, several prevalent evaluation criteria are commonly employed to assess the performance and effectiveness of the models. These evaluation criteria include the following:

#### 6.6.1. Accuracy

This criterion measures the overall correctness of predictions made by the DL model. It assesses the percentage of correct predictions compared to the total number of predictions.


$$\mathrm{Accuracy}=\frac{STN+STP}{STP+STN+SFN+SFP}*\;100$$

#### 6.6.2. Sensitivity and specificity

Sensitivity measures the ability of the model to correctly identify positive instances, while specificity measures the ability to correctly identify negative instances. These criteria are particularly relevant in medical prediction systems where correctly identifying true positives and true negatives is crucial.


$$\mathrm{Specificity}=\frac{TN}{TN+FP}*\;100$$

$$\mathrm{Sensitivity}=\frac{TP}{TP+FN}*\;100$$

#### 6.6.3. Precision and recall

Precision represents the proportion of correctly predicted positive instances out of all predicted positive instances, while recall represents the proportion of correctly predicted positive instances out of all actual positive instances. Precision and recall provide insights into the model’s ability to make accurate positive predictions and capture all positive instances.


$$\mathrm{Precision}=\frac{STP}{STP+SFP}*\;100$$

$$\mathrm{Recall}=\frac{STP}{STP+SFN}*\;100$$

F1-score: F1-score combines precision and recall into a single metric, providing a balanced evaluation of the model’s performance. It is particularly useful when dealing with imbalanced datasets or when both precision and recall are important.


$$F1‐\mathrm{score}=\frac{2*\mathrm{Recall}*P}{\mathrm{Recall}+P}*\;100$$

These evaluation criteria help researchers and practitioners assess the performance and efficacy of DL algorithms in biomedical prediction systems, providing insights into their predictive capabilities and aiding in model selection and refinement.

### 6.7. Challenges of the DL applications in biomedical prediction systems

DL applications in biomedical prediction systems face several challenges that need to be addressed to ensure their effectiveness and reliability. Some of the key challenges include:

- Data availability and quality pose significant challenges in the trends of using DL algorithms in biomedical prediction systems. Biomedical data are often limited in size due to privacy concerns and restricted access to patient records, which can hinder the training of DL models that require large datasets. Additionally, biomedical data exhibit heterogeneity across various modalities, necessitating preprocessing and normalization to ensure compatibility (Lv et al., 2022). Data quality issues such as noise, missing values, and inconsistent labeling can impact the reliability and performance of models. Furthermore, imbalanced data and ethical considerations regarding data privacy further complicate the use of DL algorithms in biomedical prediction systems. Overcoming these challenges requires standardized datasets, collaboration among stakeholders, and adherence to ethical guidelines to improve data availability and quality for robust and reliable predictions.
- Interpretability and explainability present significant challenges in the trends of using DL algorithms in biomedical prediction systems. DL models are often considered black boxes, making it difficult to understand and interpret the reasoning behind their predictions. In the biomedical field, where decision-making is critical and transparency is required, the lack of interpretability can hinder the adoption and trust in these models (Liu et al., 2023). It becomes crucial to develop techniques and methodologies that provide insights into the inner workings of DL models, enabling healthcare professionals to understand and explain the predictions. Addressing this challenge involves exploring methods for extracting meaningful features, generating explanations, and developing model-agnostic interpretability techniques to ensure that the decision-making process of DL models can be effectively understood and communicated.
- Generalization of unseen data is a significant challenge in the trends of using DL algorithms in biomedical prediction systems. DL models often excel at learning patterns and features from the training data, but their ability to generalize to new, unseen data is crucial for their real-world applicability (Wang et al., 2023). Biomedical prediction systems require models that can accurately predict outcomes for patients who were not part of the training dataset or for diseases that were not encountered during training. Overfitting, where the model becomes overly specialized to the training data and fails to generalize, is a common issue that needs to be mitigated. Techniques such as regularization, cross-validation, transfer learning, and data augmentation can help improve the generalization capability of DL models, enabling them to make reliable predictions on unseen data and novel scenarios encountered in clinical practice.
- Ethical and legal considerations pose significant challenges in the trends of using DL algorithms in biomedical prediction systems. The use of DL models in healthcare raises concerns about patient privacy, data security, and potential biases. Biomedical data often contains sensitive information, and strict regulations govern its collection, storage, and use. Adhering to ethical guidelines and privacy regulations becomes crucial to ensure the responsible and secure handling of patient data. Additionally, DL models may inadvertently perpetuate biases present in the training data, leading to unfair or discriminatory outcomes. Addressing these challenges requires implementing robust privacy protocols, data anonymization techniques, and fairness-aware algorithms to minimize biases (Xu et al., 2022). Transparency and accountability are essential in developing and deploying DL models in biomedical prediction systems to maintain patient trust, ensure compliance with legal requirements, and promote equitable and ethical use of the technology.
- Model interpretation and validation present significant challenges in the trends of using DL algorithms in biomedical prediction systems. DL models are often complex and considered black boxes, making it difficult to understand and interpret the underlying factors driving their predictions. In the biomedical field, interpretability is crucial for gaining the trust of healthcare professionals and ensuring the adoption of these models in clinical practice. Validating the performance and reliability of DL models in biomedical prediction systems requires rigorous evaluation methodologies, benchmarking against established methods, and independent validation studies. Model interpretation techniques such as attribution methods, saliency maps, or feature importance analysis can help unravel the decision-making process of DL models. Ensuring model robustness to adversarial attacks or noisy input is also important to ensure reliable performance in real-world scenarios (Shen et al., 2023). Addressing these challenges involves developing standardized evaluation protocols, promoting transparency in model architecture, and establishing best practices for model interpretation and validation in biomedical prediction systems.
- Integration with clinical workflow considerations poses a significant challenge in the trends of using DL algorithms in biomedical prediction systems. DL models need to seamlessly integrate into existing clinical workflows and healthcare systems to facilitate their practical deployment and adoption by healthcare professionals. However, clinical workflows often have specific requirements, data formats, and compatibility constraints that may not align with the characteristics of DL models. Integration challenges may include data interoperability, integration with EHR systems, real-time prediction capabilities, and the need for user-friendly interfaces (Zeng et al., 2020). Close collaboration among researchers, healthcare professionals, and technology experts is necessary to understand the workflow requirements, identify integration points, and develop solutions that harmonize the capabilities of DL models with the clinical setting. Ensuring smooth integration with clinical workflows is vital to maximize the utility and effectiveness of DL algorithms in biomedical prediction systems and to facilitate their practical implementation in real-world healthcare environments (Liu et al., 2021; Qi et al., 2022; Yang et al., 2022).

### 6.8. Biases and impurities involved in DL algorithms used for biomedical prediction systems

In the application of CNNs in biomedical prediction systems, impurities encompass extraneous elements or characteristics within the data. This can manifest as noise, comprising irrelevant or random fluctuations in the biomedical data that do not contribute meaningful information (Wang et al., 2021). Furthermore, impurities may arise from artifacts in the data acquisition process, inconsistencies in labeling, or even the presence of outliers. On the other hand, inherent biases pertain to systematic inclinations in CNN’s learning process that could lead to skewed predictions. In the biomedical context, biases can stem from imbalances in class distribution, where certain medical conditions may be overrepresented or under-represented, potentially influencing the model’s performance and generalization to diverse patient populations. Researchers and practitioners must be aware of and address these impurities and biases to ensure the reliability and effectiveness of CNN-based predictive models in biomedical applications (Zhang et al., 2023).

In the realm of utilizing RNNs for biomedical prediction systems, impurities denote unwanted elements or characteristics present in the data. These can encompass noisy fluctuations or irrelevant features within the biomedical data, as well as potential artifacts from data collection or labeling inconsistencies. Additionally, impurities may arise from outliers or anomalies in the dataset, which can potentially impact the RNN’s ability to generalize effectively (Shan et al., 2023). Inherent biases, on the other hand, refer to systematic tendencies within the RNN’s learning process that could lead to skewed predictions. This might be especially pertinent in biomedical applications due to imbalances in class distribution, where certain medical conditions may be disproportionately represented, potentially influencing the model’s performance on under-represented conditions. Being cognizant of and mitigating these impurities and biases is crucial for ensuring the accuracy and robustness of RNN-based predictive models in biomedical contexts (Cheng et al., 2017).

In the context of trends in using DL algorithms in biomedical prediction systems, when employing GANs, impurities refer to unwanted elements or characteristics within the data. These can arise from various sources, such as noise or irrelevant features in biomedical datasets, as well as potential inconsistencies or inaccuracies in data labeling. Moreover, impurities might result from outliers or anomalies that could affect the GAN’s ability to generate realistic and representative samples. Inherent biases, on the other hand, pertain to systematic tendencies in the GAN’s learning process that may lead to skewed outputs (Tang et al., 2021). In the biomedical domain, this could be influenced by imbalances in class distribution, potentially causing the GAN to overrepresent certain conditions or features. Recognizing and addressing these impurities and biases are vital for ensuring the reliability and effectiveness of GAN-based predictive models in biomedical applications (Zhang et al., 2022).

Impurities are undesired aspects or features present in the data, as discussed in trends in using DL algorithms in biomedical prediction systems while discussing the use of LSTM networks. Biomedical datasets may contain noisy or pointless characteristics, as well as potential data collecting errors or inconsistent labeling (Lu et al., 2023). Additionally, impurities might result from outliers or abnormalities in the data, which can impair the LSTM’s capacity to detect important temporal correlations. On the other hand, inherent biases are systematically ingrained tendencies inside the LSTM’s learning process that could result in distorted predictions. In the biological field, this may be impacted by unequal distribution of classes, which might make the model better at foretelling common disorders or patterns. For LSTM-based prediction models to be accurate and robust in biomedical applications, these biases and impurities must be identified and minimized (Liu et al., 2023).

When used in the context of SVM, impurities refer to undesired features or qualities in the data that may be present in biomedical datasets as noise, pointless features, or outliers and may prevent the SVM from drawing precise decision lines. Inconsistencies in data labeling or artifacts created during the data collection process may also result in contaminants. In contrast, intrinsic biases are routinely recurring tendencies during the SVM learning process that may result in erroneous predictions. This might happen in the biomedical area because of imbalances in the distribution of class labels, where specific medical ailments may be over- or under-represented in the dataset, hurting the model’s performance for less prevalent disorders (Dang et al., 2023).

In the realm of using DL algorithms in biomedical prediction systems, both the quality of data and the sophistication of the employed model play pivotal roles. High-quality, diverse, and well-curated datasets are the cornerstone of robust predictive models, enabling them to learn intricate patterns and relationships within the biomedical domain. However, the complexity of biomedical data often demands sophisticated models capable of capturing non-linear dependencies and extracting intricate features. CNNs excel in image-related tasks, such as medical imaging analysis, due to their adeptness in hierarchically learning features (Sarbaz et al., 2022). RNNs and their variants, on the other hand, are adept at processing sequential data, making them suitable for tasks such as time-series analysis in healthcare. Furthermore, attention mechanisms and transformer-based architectures have gained prominence for tasks requiring contextual understanding and long-range dependencies. Additionally, transfer learning techniques, where models pre-trained on large-scale datasets are fine-tuned for specific biomedical tasks, have shown promise in mitigating data scarcity issues (Bagheri et al., 2020). The integration of ensemble methods and techniques such as Bayesian DL further enhances model robustness and uncertainty quantification, vital in clinical decision-making contexts. Striking the right balance between data quality and model sophistication is crucial, as overly complex models without adequate data may lead to overfitting, while inadequate models may struggle to capture the intricacies of biomedical information. Hence, an informed selection of both data and model architecture is paramount in achieving accurate and reliable predictions in biomedical applications (Soleimani and Lobaton, 2022).

### 6.9. Addressing data labeling, variable filtering and selection, class distribution, or potential embeddings

In the reviewed works on the application of CNNs in biomedical prediction systems, several key considerations were addressed to optimize model performance. Data labeling was meticulously conducted, leveraging expert knowledge to ensure accurate annotations of medical images or signals. Variable filtering and selection techniques were employed to extract salient features, reducing noise and irrelevant information while preserving critical biomarkers (Ahmadi and Khotanlou, 2022). Class distribution imbalances, a common issue in medical datasets, were addressed through techniques such as data augmentation, stratified sampling, or advanced loss functions, ensuring that the model learned from all classes equally. Furthermore, potential embeddings, such as pre-trained CNN architectures or domain-specific embeddings, were leveraged to initialize the network weights, enabling the model to learn robust representations even with limited data. By systematically tackling these challenges, the reviewed works demonstrated the effectiveness of CNNs in extracting meaningful information from biomedical data for accurate prediction and diagnosis. In addition, in the examined studies focusing on the application of RNNs in biomedical prediction systems, several critical strategies were employed to enhance model efficacy. Data labeling was conducted with meticulous attention to detail, involving domain experts to ensure precise annotations of sequential medical data (Morteza et al., 2023). Variable filtering and selection techniques were applied to extract relevant features, effectively reducing noise and irrelevant information while preserving essential biomarkers. To address potential class distribution imbalances, which are prevalent in medical datasets, researchers utilized methods such as oversampling, undersampling, or class-weighted loss functions, ensuring that the model learned from all classes equitably. Additionally, potential embeddings, such as pre-trained RNN architectures or domain-specific embeddings, were leveraged to initialize the network weights, enabling the model to learn robust representations from the sequential data. These comprehensive approaches collectively showcased the effectiveness of RNNs in discerning meaningful patterns and relationships within biomedical data, leading to accurate predictions and diagnoses. In the scrutinized studies focused on utilizing GANs in biomedical prediction systems, specific strategies were employed to optimize model performance. Data labeling was executed meticulously, often involving expert annotations to ensure accurate labeling of medical images or signals (Webber et al., 2017). Variable filtering and selection techniques were applied to extract salient features, reducing noise and irrelevant information while retaining critical biomarkers. Addressing class distribution imbalances, common in medical datasets, researchers utilized techniques such as generative oversampling or incorporating additional loss terms to balance the learning process. Moreover, potential embeddings, such as using pre-trained GAN architectures or domain-specific embeddings, were leveraged to initialize network weights, allowing the model to learn robust representations from limited data. These comprehensive approaches collectively demonstrated the efficacy of GANs in extracting meaningful information from biomedical data for accurate prediction and diagnosis. In the examined works concerning the utilization of LSTM networks in biomedical prediction systems, several key methodologies were adopted to enhance model performance (Webber et al., 2022). Data labeling was conducted meticulously, often with the involvement of domain experts, to ensure precise annotations of sequential medical data. Variable filtering and selection techniques were employed to extract relevant features, reducing noise and extraneous information while retaining vital biomarkers within the sequential data. Class distribution imbalances, a common challenge in medical datasets, were addressed through techniques such as resampling or class-weighted loss functions, ensuring equitable learning from all classes. Furthermore, potential embeddings, such as initializing LSTM weights with pre-trained models or domain-specific embeddings, were utilized to facilitate the network’s capacity to capture meaningful patterns within the sequential data. These comprehensive approaches collectively showcased the effectiveness of LSTM networks in extracting valuable insights from biomedical data for accurate prediction and diagnosis (Kosarirad et al., 2022). In addition, in the reviewed works, focusing on the SVM method in biomedical prediction systems, distinct strategies were implemented to optimize model performance. Data labeling was conducted meticulously, often involving domain experts to ensure precise annotations of biomedical features. Variable filtering and selection techniques were applied to extract relevant features, reducing noise and irrelevant information while preserving critical biomarkers. Addressing class distribution imbalances, which are prevalent in medical datasets, researchers employed techniques such as cost-sensitive learning or resampling methods to mitigate biases in the learning process. While SVM is a more traditional ML algorithm compared to DL methods, the attention to data quality, feature selection, and class balance remains crucial for its effectiveness in biomedical predictions. These comprehensive approaches collectively demonstrated the efficacy of SVMs in discerning meaningful patterns and relationships within biomedical data, leading to accurate predictions and diagnoses (Gera et al., 2021).

### 6.10. Dataset in biomedical prediction system

In the context of trends in using DL algorithms in biomedical prediction systems, the dataset refers to the collection of data used for training, validating, and testing DL models. The dataset plays a crucial role in the development and evaluation of these models (Moradi et al., 2020). Biomedical datasets encompass a wide range of data types, including medical images, EHRs, genomic sequences, clinical notes, and physiological signals. These datasets are often complex and diverse, and can vary in size, quality, and annotation availability. They serve as the foundation for training DL models to learn patterns, extract features, and make predictions related to various biomedical applications, such as disease diagnosis, prognosis, treatment response prediction, and personalized medicine (Bukhari et al., 2022). Constructing and curating high-quality, representative, and well-annotated datasets are essential to ensure the effectiveness, generalization, and ethical use of DL algorithms in biomedical prediction systems.

In this context, several datasets are commonly used for research and development. Some notable datasets include the following:

- MIMIC-III (Medical Information Mart for Intensive Care III): This dataset consists of de-identified health records from ICU patients, including demographics, vital signs, laboratory measurements, medications, and clinical notes. It is widely used for developing predictive models in critical care settings.
- ImageNet: While not specific to biomedical data, ImageNet is a large-scale dataset containing millions of labeled images across various categories. It has been used to pre-train DL models for transfer learning in medical image analysis tasks.
- TCGA (The Cancer Genome Atlas): TCGA is a comprehensive dataset that provides genomic, transcriptomic, and clinical data for various cancer types (Zemouri et al., 2018). It enables researchers to develop DL models for cancer diagnosis, prognosis, and therapeutic response prediction.
- CheXpert: This dataset focuses on chest radiographs and provides labeled images with associated radiologist interpretations. It is commonly used for training DL models for chest X-ray interpretation and disease detection, such as pneumonia and lung nodules.
- PhysioNet: PhysioNet offers a collection of datasets for various physiological signals, such as electrocardiography (ECG), electroencephalography (EEG), and blood pressure. These datasets facilitate the development of DL models for signal analysis and disease detection.

These datasets, among others, serve as valuable resources for researchers and practitioners in the field of biomedical prediction systems, allowing them to explore and advance DL algorithms in healthcare applications (Momeni et al., 2020).

Datasets are pivotal in utilizing DL algorithms for biomedical prediction systems. They form the basis for training and evaluating DL models, enabling them to extract meaningful patterns from complex healthcare data. These datasets empower a wide range of tasks, from disease diagnosis to treatment response prediction. Diverse and representative datasets enhance model generalization and applicability across different contexts (Singh et al., 2022). They also enable benchmarking and collaboration, advancing biomedical prediction systems. However, challenges arise, including the limited availability of high-quality data due to privacy regulations and expertise requirements. Heterogeneity in data formats and quality, as well as potential biases and errors, necessitate careful preprocessing and integration efforts. Ethical considerations surrounding patient privacy mandate secure data handling practices. Overcoming these challenges requires standardized protocols, collaborative efforts, and innovative techniques for data augmentation and quality control in biomedical prediction systems (He et al., 2022).

### 6.11. Security issues, challenges, risks, IoT, and blockchain usage

The use of DL algorithms in biomedical prediction systems raises significant security and risk considerations. Healthcare data, such as patient records and genetic information, are vulnerable to breaches, making robust security measures essential (Jafari et al., 2022). DL models can also be susceptible to adversarial attacks, further emphasizing the need for data protection and secure deployment in healthcare settings. However, there are inherent risks, including potential incorrect predictions, biases, and overfitting of models. These issues can impact patient care and introduce ethical concerns. Balancing innovation and patient safety requires stringent evaluation, regulatory oversight, and continuous monitoring of DL algorithms in biomedical prediction systems. The need for resilience and dependable management that has been generalized in this context is critical (Amiri et al., 2023).

Underfitting in the context of using DL algorithms in biomedical prediction systems represents a critical challenge (Esmaeili and Bamdad Soofi, 2022). It occurs when the chosen model is too simplistic to capture the underlying complexity of the biomedical data, resulting in poor predictive performance. In such cases, the model fails to learn the intricate patterns and relationships within the data, leading to a significant gap between predicted and actual outcomes. This phenomenon is particularly detrimental in biomedical applications, where accurate predictions are paramount for informed clinical decisions. To mitigate underfitting, researchers must carefully select and architect models that have sufficient capacity to grasp the complexities of the underlying biological processes (Sadi et al., 2022). Additionally, techniques such as fine-tuning, transfer learning, and model assembling can be employed to enhance the model’s capacity to learn from the data effectively. Balancing model complexity with the richness of the available data is crucial to achieving optimal predictive performance in biomedical applications.

The adoption of DL algorithms in biomedical prediction systems brings potential risks that must be addressed. These include the possibility of incorrect predictions impacting patient care, biases, and overfitting issues (Rastegar et al., 2022). Ethical concerns arise regarding privacy breaches and the perpetuation of existing healthcare disparities. Blockchain technology emerges as a promising solution to enhance security and privacy in these systems. Its decentralized, immutable nature ensures data integrity and privacy, offering a secure platform for managing healthcare data. By implementing blockchain, DL algorithms can access and analyze data from various sources while upholding patient privacy (Moradi et al., 2022). Smart contracts enable secure data-sharing agreements, fostering collaboration among stakeholders. This integration enhances data governance, encourages sharing, and builds trust, ultimately leading to more reliable DL models in healthcare.

## 7. Open issues

The use of DL algorithms in biomedical prediction systems holds promise but presents several challenges. Interpretability and explainability are crucial, given the complexity of DL models (Khorshidi et al., 2023). Addressing these concerns is vital for transparency and trust. Data scarcity and quality are ongoing hurdles, particularly in the diverse and imbalanced biomedical data landscape. Standardizing data collection and sharing protocols, along with augmentation techniques, can help mitigate these challenges. Ethical considerations, including privacy protection and algorithmic bias, are paramount (Esmaeili and Bamdad Soofi, 2022). Balancing data access for research while safeguarding privacy is key. Integrating DL into clinical workflows and systems requires seamless compatibility with existing infrastructure, real-time capabilities, and robust security measures. Rigorous evaluation methods are essential for establishing the reliability of DL models in real-world clinical settings. Collaborative efforts are needed to overcome these challenges and ensure the responsible use of DL algorithms in biomedical prediction systems (Wu et al., 2023).

### 7.1. Key research challenges

The trends involving the use of DL algorithms in biomedical prediction systems bring forth several key research challenges. These challenges are essential to address to advance the field and maximize the potential of DL in healthcare.

Key research challenges in the application of DL models to biomedical prediction systems include the need for interpretability and explainability, especially in contexts where transparency and trust are crucial. Efforts are underway to develop explainable models and visualization techniques to shed light on the decision-making process (Esmaeili and Bamdad Soofi, 2022). Another challenge lies in the availability and quality of data, with researchers exploring techniques such as data augmentation and transfer learning. Standardizing data protocols is seen as a way to enhance data quality. Ethical considerations, such as privacy protection and bias mitigation, are significant concerns, with ongoing efforts to develop privacy-preserving methods and fairness-aware algorithms. Integrating DL algorithms into clinical workflows, ensuring real-time capabilities, and interoperability are also key challenges, as is establishing rigorous evaluation methodologies. Collaborative efforts are needed to address these challenges and fully realize the potential of DL in biomedical prediction systems (Li et al., 2023).

DL architectures have witnessed a transformative shift with the integration of powerful inductive biases. Transformers, exemplifying this paradigm, have gained prominence for their adeptness in tasks involving sequential data, thanks to their attention mechanisms (Mirzapour and Arpanahi, 2017). These mechanisms allow the model to weigh the importance of different elements in the input, enabling it to capture long-range dependencies effectively. This innovation has revolutionized applications such as natural language processing, where understanding contextual relationships is paramount. Simultaneously, graph-based models, particularly graph neural networks (GNNs), have emerged as a pivotal trend. GNNs are tailored to handle data with intricate relational structures, such as social networks or molecular graphs. By encoding information about node neighborhoods, they excel in tasks such as node classification, link prediction, and graph-level predictions. This trend reflects a pivotal shift toward architectures that can adeptly model complex relationships and dependencies, signifying the dynamic evolution of DL research to adapt to a diverse range of data modalities (Zhang et al., 2019).

### 7.2. Systematic standardization in biomedical measurement protocols

Systematic standardization of biomedical measurement protocols is a critical aspect in ensuring the reliability and reproducibility of research outcomes. In the context of employing DL algorithms for biomedical prediction systems, standardization challenges arise from variations in data acquisition, preprocessing, and feature extraction methods across different studies. These discrepancies can lead to inconsistent results and hinder the generalizability of predictive models (Shen et al., 2017). To address these challenges, the literature emphasizes the importance of establishing rigorous and well-documented protocols for data collection, including the use of standardized instruments and procedures. Additionally, preprocessing steps such as data normalization and augmentation are crucial to mitigate biases and enhance model performance. Furthermore, the incorporation of domain expertise and collaborations among clinicians, data scientists, and engineers is advocated to ensure that the chosen protocols align with clinical best practices (Ahmadi and Khotanlou, 2017). Moreover, the adoption of open-access datasets and the publication of code and models facilitate transparency and reproducibility, enabling the broader scientific community to validate and build upon existing research findings. By systematically addressing standardization issues, researchers can enhance the robustness and applicability of DL algorithms in biomedical prediction systems, ultimately advancing the field toward more reliable and clinically relevant outcomes (Amiri et al., 2023).

### 7.3. Deeper discussion on the challenges associated with the use of subjective data

The utilization of subjective data in biomedical prediction systems presents a multifaceted challenge rooted in the inherently qualitative and context-dependent nature of such information (Ahmadi and Khotanlou, 2022). Unlike objective measurements, subjective data relies on individual perceptions, interpretations, and self-reported experiences, introducing a level of variability and potential bias that can impede algorithmic performance. Subjective data can encompass a wide range of inputs, including patient-reported outcomes, qualitative assessments by healthcare professionals, and even crowd-sourced information from online platforms. This diversity further complicates the development of standardized protocols for preprocessing and integrating subjective data into predictive models (Shen et al., 2018). Additionally, ensuring the reliability and consistency of subjective data is paramount, as factors such as cultural background, emotional state, and communication skills can influence the accuracy of self-reported information. Moreover, the integration of subjective data requires a nuanced approach to feature selection and representation, as traditional quantitative techniques may not adequately capture the subtleties inherent in subjective assessments (Rezaei et al., 2023). Addressing these challenges necessitates interdisciplinary collaboration between clinicians, psychologists, data scientists, and domain experts, as well as the development of specialized techniques within DL frameworks to effectively extract and interpret subjective information. By doing so, researchers can unlock the full potential of subjective data in biomedical prediction systems, enabling more comprehensive and personalized healthcare solutions, especially for ML techniques in the Internet of Behaviors (Amiri et al., 2023).

### 7.4. Future works

In the future, trends involving the use of DL algorithms in biomedical prediction systems will likely focus on several areas of advancement. These future works will aim to address existing challenges, improve performance, and expand the scope and impact of DL in healthcare.

Future work in the application of DL models to biomedical prediction systems will focus on several key areas. First, there will be a concerted effort to enhance the interpretability and explainability of these models, potentially through the development of new attention mechanisms and visualization techniques. Second, researchers will work toward creating more robust and comprehensive datasets specific to various biomedical prediction tasks, addressing data heterogeneity and ensuring data quality (Dehghani and Larijani, 2023b; Han and Fu, 2023). Ethical considerations will remain a central focus, with the development of frameworks to address privacy, security, fairness, and transparency. Integration into clinical workflows and healthcare systems will also be a priority, requiring the seamless incorporation of DL algorithms into existing infrastructure. Finally, there will be a push for rigorous evaluation and validation frameworks to ensure the reliability and safety of DL models in clinical practice (Dehghani and Larijani, 2023a). Collaboration between researchers, clinicians, industry experts, and regulatory bodies will be essential to drive progress in these areas and ultimately improve patient care and healthcare outcomes.

## 8. Conclusion and limitations

This review article highlights the significant advancements and trends in utilizing DL algorithms for biomedical prediction systems. The extensive analysis of various algorithms, such as CNN, RNN, GAN, LSTM, SVM, and hybrid methods, showcases their roles and contributions in addressing the challenges of biomedical prediction. The discussed advantages of these methods, including their ability to handle complex data, extract meaningful features, and achieve high prediction accuracy, demonstrate their potential for revolutionizing biomedical research and healthcare applications. However, it is important to acknowledge the existing challenges, such as data scarcity, interpretability, and computational requirements, which require further attention and research efforts. Nonetheless, the field is continuously evolving, and further research is necessary to explore new techniques and methodologies that can enhance the performance and robustness of DL algorithms in the context of medical and health prediction systems. In addition, there is a need for more comprehensive evaluations of DL algorithms in real-world scenarios and for the development of robust and scalable systems that can be deployed in healthcare settings. Therefore, it is imperative to continue conducting research in this area to fully leverage the potential of DL in image segmentation in medical healthcare and provide better healthcare outcomes for patients. Initially, we discuss the advantages and disadvantages of some surveyed articles regarding this investigation, before illustrating the strategy of this article. The medical prediction systems platforms and tools are also assessed. Based on a survey of articles according to qualitative features, most articles are assessed relying on accuracy, sensitivity, specificity, F-score, adaptability, scalability, and precision. However, certain features, such as security and convergence time, are underutilized. To evaluate and perform the proposed methods, various programming languages are used, where Python is the most commonly used in related studies. Furthermore, we anticipate that our investigation will provide a valuable guide for further research on DL-based methods in medical and health prediction systems issues. Nevertheless, some constraints were encountered during our analysis, including the unavailability of non-English-language articles, which limited our ability to utilize numerous investigation initiatives. In addition, since this field is still relatively new, there was a limited number of published research works specifically focusing on this topic. Consequently, there was a scarcity of high-quality research articles, which impacted the comprehensiveness and depth of the review. In addition, the heterogeneity in methodologies, datasets, and evaluation metrics employed across the included studies posed challenges in synthesizing the findings and drawing conclusive insights. The variability in study designs and approaches also limited the ability to perform quantitative meta-analysis, further restricting the level of evidence that could be derived. Furthermore, the rapidly evolving nature of DL techniques and the emergence of new algorithms and architectures might render the study’s findings relatively time-sensitive, potentially requiring frequent updates or revisions to remain up-to-date. Finally, a shortage of articles published by renowned publications was another limitation we faced. Despite these limitations, DL has emerged as a game-changing approach for developing intelligent solutions to complex problems, and we hope that the results of this study will aid in the advancement of DL approaches on real-world platforms. All in all, this review article provides valuable insights into the current state and future directions of DL algorithms in biomedical prediction systems, serving as a valuable resource for researchers and practitioners in the field.

## Data availability statement

The original contributions presented in the study are included in the article/supplementary material, further inquiries can be directed to the corresponding author.

## Author contributions

YW: Investigation, Writing – original draft, Writing – review & editing. LL: Investigation, Writing – original draft, Writing – review & editing. CW: Investigation, Supervision, Writing – original draft, Writing – review & editing.
